# Toward Scalability: Fe‐MOF‐Based Ultrafiltration Membrane for Effective Microplastics Removal from Drinking Water at Point‐of‐Use

**DOI:** 10.1002/gch2.202500559

**Published:** 2026-01-07

**Authors:** Sahil Shrestha, Ajaya Subedi, Shane A. Snyder, Michael J. Angove, Shukra Raj Paudel

**Affiliations:** ^1^ Environmental Engineering Program Department of Civil Engineering Pulchowk Campus Institute of Engineering Tribhuvan University Pulchowk, Lalitpur Nepal; ^2^ School of Civil and Environmental Engineering College of Engineering, Georgia Institute of Technology Atlanta Georgia USA; ^3^ Nanyang Environment Water Research Institute (NEWRI) Nanyang Technological University Singapore Singapore; ^4^ Department of Rural Clinical Sciences La Trobe Rural Health School La Trobe University Australia

**Keywords:** membrane filtration, metal‐organic frameworks, microplastics, point‐of‐use, scalability

## Abstract

The frequent detection of microplastics (MPs) in bottled drinking water underscores the need for effective point‐of‐use (POU) purification strategies to limit human exposure, particularly given their ability to transport co‐contaminants. While metal‐organic frameworks (MOFs) have been extensively investigated for MP removal, their application in practical POU drinking water purification remains largely underexplored, especially regarding scalability and delivery of potable water after filtration. In this work, NH_2_‐MIL‐101(Fe) MOF is integrated onto a commercial polyvinylidene fluoride (PVDF) ultrafiltration (UF) membrane to develop a Fe‐MOF@UF composite for enhanced removal of polyethylene terephthalate (PET)‐MP from drinking water. The optimally synthesized Fe‐MOF@UF membrane achieved a PET‐MP rejection efficacy of ∼94%. Additionally, its practical applicability is validated using commercially available PET‐bottled drinking water, confirming the effective removal of MPs while delivering potable water compliant with international drinking water quality standards. Collectively, these outcomes emphasize the first practical viability of MOF‐membrane hybrids for POU drinking water treatment. Despite limitations, this research lays a strong groundwork for future efforts toward performance optimization and highlights a viable pathway for scalable, cost‐effective, and sustainable MOF‐incorporated household MP filtration units.

## Graphical abstract

This study designs a unique Fe‐MOF@UF composite membrane for point‐of‐use drinking water purification, targeting microplastic removal. The system is fundamentally optimized and validated with commercial bottled‐water samples, achieving safe potable water production. Findings establish the first practically viable MOF‐membrane hybrid and emphasize its scalability toward sustainable and user‐friendly application in commercial drinking water purification units.

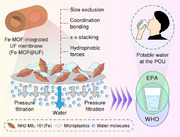

## 1 Introduction

Global plastic production has escalated sharply due to its widespread use in textiles, cosmetics, and packaging [1, 2], surpassing 9000 million metric tons (Mt) of cumulative plastic production as of 2024 [3]. However, it is worth noting that in 2023, only about 9% of the 414 Mt of plastics consumed were recycled, leaving approximately 375 Mt to accumulate as discarded plastic waste [4]. Owing to their durable, long‐chain polymeric structures, these plastics resist immediate degradation and instead persist in the environment for centuries. Over time, they fragment into micro‐sized particles, popularly known as microplastics (MPs)—particles that typically range below 5 mm in size [5, 6]. These MPs originate either as primary forms, such as cosmetic microbeads and synthetic fibers/pellets used in manufacturing plastic products, that are deliberately produced, or as secondary forms generated through the environmental fragmentation of larger plastic debris via prolonged physicochemical and biological processes [7, 8, 9]

MPs originating from various polymers are now globally pervasive, spanning deep‐sea habitats to commercial drinking water [10]. Among these, commercial bottled drinking waters, particularly those packaged in single‐use or reused Polyethylene Terephthalate (PET) bottles, are widely reported to contain PET‐MPs [11, 12]. Multiple factors contributing to such contamination include high‐pressure cleaning processes, abrasion during manufacturing, leaching from plastic walls under thermal stress, and consumer handling practices like shaking or compressing bottles [13]. Consequently, as bottled water consumption rises globally, so does the human exposure. As a result, MPs are ingested into the human body, particularly corroborated by their detection within various bodily systems, including the central nervous, skeletal, and reproductive systems [14, 15]. Furthermore, MP's large surface area enables them to adsorb and transport hazardous contaminants and pathogens [16]. Therefore, these observations underscore the urgent need for effective point‐of‐use (POU) drinking water purification strategies—particularly targeting PET‐MPs—to protect public health.

Commercial membrane filtration technologies, such as microfiltration (MF), ultrafiltration (UF), nanofiltration (NF), and reverse osmosis (RO), are extensively used in household POU filtration units. These methods primarily rely on size exclusion to remove contaminants, including MPs. While these standalone membranes have demonstrated promising MP removal efficiency [17, 18], they are significantly prone to fouling due to the minute MPs entering membrane pores [19]. Specifically, prolonged filtration of MP‐laden water leads to the surface accumulation and pore blockage, which elevate transmembrane pressure, diminish water flux, and increase overall energy demand [20]. While backwashing can alleviate fouling, it remains largely impractical in most household filtration units due to inherent design constraints and the need for specialized operation [21]. Consequently, end‐users typically rely on periodic filter replacement as prescribed by manufacturers to sustain membrane performance. Thus, to address these issues, surface modification of membranes with effective adsorbents is essential for desirable MP rejection [22, 23], while ensuring an overall user‐friendly household filtration apparatus.

Among advanced adsorbents developed, metal‐organic frameworks (MOFs) have garnered significant attention in recent years due to their demonstrated ability to remove MPs, enhancing water purification. MOFs are porous crystalline materials composed of metal ions intricately linked with organic ligands, forming coordinated polymeric networks [24, 25]. They exhibit excellent performance due to their high surface area, tunable porosity, recyclability, and hydrophilicity [26]. Studies have also explored their MP removal efficiencies in particulate forms [22]. However, due to challenges in material handling and the risk of accidental environmental release, their application is restricted to lab‐based experiments rather than real‐world treatment. To overcome this, researchers have developed MOF‐membrane composite filtration systems, wherein MOFs are incorporated into or coated onto membrane substrates to enhance stability and filtration performance. For example, Golgoli et al. [27] modified a thin film composite FO membrane with MIL‐53 (Fe)‐based MOFs to improve its MP fouling resistivity. Gnanasekaran et al. [28]. incorporated MIL‐100 (Fe) MOF particles into a polysulfone (PSF) membrane matrix for MP removal from wastewater. In another work, Chen et al. [29]. developed a melamine foam reinforced with Zirconium‐based MOFs for efficient MP removal from simulated suspensions. Nonetheless, most existing studies on MOF‐membrane composites have primarily focused on MP removal from the wastewater domain, whereas their application for drinking water purification remains largely underexplored, representing a critical research gap. Although processed bottled and jar drinking waters generally contain lower MP concentrations than wastewater or natural waters [13, 30], continuous uptake of such MP‐contaminated water still poses a tangible public health risk. Therefore, this emphasizes the necessity for practical, scalable MOF‐integrated hybrid membranes capable of removing MPs and delivering potable water at the POU, particularly for household/commercial drinking water purification units. Furthermore, to date, no literature has evaluated the water potability following treatment with MOF‐integrated membranes, highlighting both the novelty and the rationale of this study.

To address these challenges and mitigate MP contamination in drinking water at the POU, this study engineered an innovative Fe‐MOF@UF composite membrane by integrating NH_2_‐MIL‐101(Fe) MOF into a 4‐inch commercial Polyvinylidene Fluoride (PVDF)‐based UF membrane. The system was systematically optimized and evaluated for PET‐MP removal, including assessments of synthesis scenarios, operating conditions, and reusability. Validation using real commercial PET‐bottled water demonstrated improved filtrate quality compared to bare UF membranes, affirming both the scalability potential of the system and the potability of the treated water in accordance with international drinking water guidelines. Importantly, this research represents the first demonstration of a MOF‐membrane composite for practical POU drinking water purification, combining enhanced MP removal, scalability, and potable water assurance. While further investigations are warranted to broaden applicability, the findings of this work provide a robust framework for the future development of advanced, scaled‐up MOF‐based membranes, aiming to reduce human exposure to MPs while supporting efforts to achieve Sustainable Development Goal 6 (Clean Water and Sanitation).

## 2 Results and Discussion

### 2.1 Material Characterization

**FIGURE 1:**
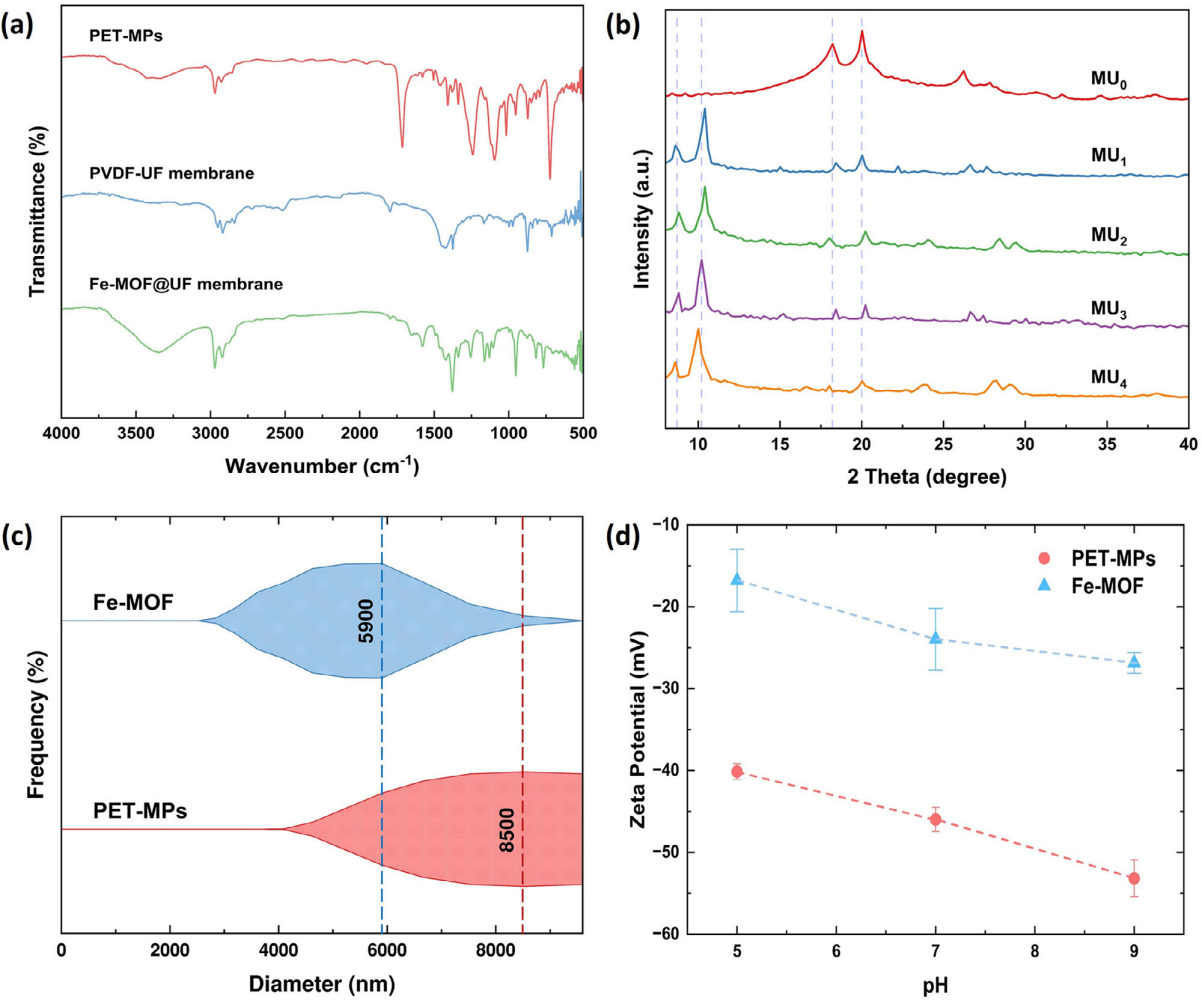
Physicochemical characterization of materials. (a) FTIR spectra illustrate the characteristic absorption bands of PET‐MPs, pristine PVDF‐UF membrane, and synthesized Fe‐MOF@UF membrane. (b) XRD diffraction patterns highlight the primary peaks representing corresponding crystal structures of the bare PVDF‐UF membrane (MU_0_) and the Fe‐MOF@UF membranes (MU_1_ to MU_4_). (c) Kite diagram demonstrate the DLS‐derived particle size distributions of Fe‐MOF and PET‐MPs, where kite widths indicate frequency and dashed lines denoting mean diameters of the particles. (d) Zeta potential profiles showcase that both Fe‐MOF particles (closed blue triangle) and PET‐MPs (closed red circles) remain negatively charged across varying pH.

The FTIR spectra in Figure 1a validated the characteristic functional groups of the collected MP particles, pristine UF membrane, and Fe‐MOF@UF membranes. The MP particles exhibited absorption peaks at 2970–2929, 1714, 1240–1096, 872, and 723 cm^−1^, corresponding to the typical PET functional groups, thereby affirming their polymeric identity [31, 32]. The bare UF membrane displayed distinct bands at 1427–1375, 1170, 972, and 844 cm^−1^, consistent with typical vibrational features of PVDF [33, 34]. Moreover, new peaks emerged at 3345, 1658, 1578–1377, 1255, and 767–559 cm^−1^ upon Fe‐MOF incorporation, collectively validating the successful integration of NH_2_‐MIL‐101(Fe)‐MOF over the UF membrane [35, 36]. This spectral analysis highlights the possibility of hydrogen bonding interactions between the carboxylate (C = O) groups of Fe‐MOF (1578‐1377 cm^−1^) and the electronegative fluorine atoms of PVDF's ‐CF_2_ groups (1170 cm^−1^). Concurrently, the strongly polar ‐NH_2_ groups of Fe‐MOF (3345 cm^−1^) may also induce temporary dipoles in the ‐CH_2_ molecules of PVDF membrane (1375–1427 cm^−1^), indicative of London‐dispersion forces via dipole‐induced dipole interactions. Therefore, these interactions likely promote the physical anchoring of Fe‐MOF crystals over the rough PVDF membrane surface (Figure 5). Furthermore, these spectra highlight the surface chemistries that are likely responsible for facilitating interactions with MPs and contributing to their removal. The detailed vibrational modes assignments are provided in Table S1.

Figure 1b illustrates the XRD patterns of the unmodified PVDF‐based UF membrane (MU_0_) and the Fe‐MOF@UF membranes (MU_1_ to MU_4_)—each synthesized with varying MOF dosages (Table S2). The bare membrane displayed distinct diffraction peaks at 18.21° (020) and 20.02° (110)—characteristic of PVDF's α‐phase crystallinity [37]—confirming the PVDF polymer of the procured UF membrane. In contrast, Fe‐MOF@UF membranes presented two prominent Fe‐MOF peaks at ∼8.7° (002) and ∼10.2° (101), matching the signature reflections of NH_2_‐MIL‐101(Fe)‐MOF particles [35]. Further, the suppressed peaks at around 18° and 20° in the same spectra possibly signified the PVDF membrane substrate supporting these MOF particles, thereby confirming the successful integration of Fe‐MOF crystals over the UF membrane substrate.

The mean hydrodynamic diameters and zeta (ζ) potentials of PET‐MPs and NH_2_‐MIL‐101(Fe)‐MOF across pH 5, 7, and 9 are demonstrated in Figure 1c,d. The particle size distribution of the procured PET‐MPs (Figure 1c) verified their classification as MPs (< 1 mm). Likewise, the synthesized MOF particles exhibit characteristic microscopic dimensions, likely regulated by synthesis conditions, such as the adopted reagent concentrations and reaction period. The ζ‐potential of PET‐MPs remained consistently negative throughout the tested pH range (Figure 1d). This trend aligns with literature illustrating that increasing pH enhances negative surface charge of such a common polymer, primarily due to dissociation of intrinsic carboxyl (–COOH) groups in PET's structure [38]. Across the same pH range, the MOF particles also exhibited negative ζ‐potentials with rising pH, presumably attributed to the progressive deprotonation of amine (‐NH_3_^+^) groups in the NH_2_‐MIL‐101(Fe)‐MOF structure [39]. Nonetheless, slight variations from reported values may stem from differences in synthesis protocols. Despite both materials bearing negative surface charges, combined interactions between MOF and PET‐MP particles—such as coordination bonding, π‐π stacking, and hydrophobic interactions—can still occur, promoting enhanced MP capture during filtration, as elaborated in subsequent sections.

**FIGURE 2:**
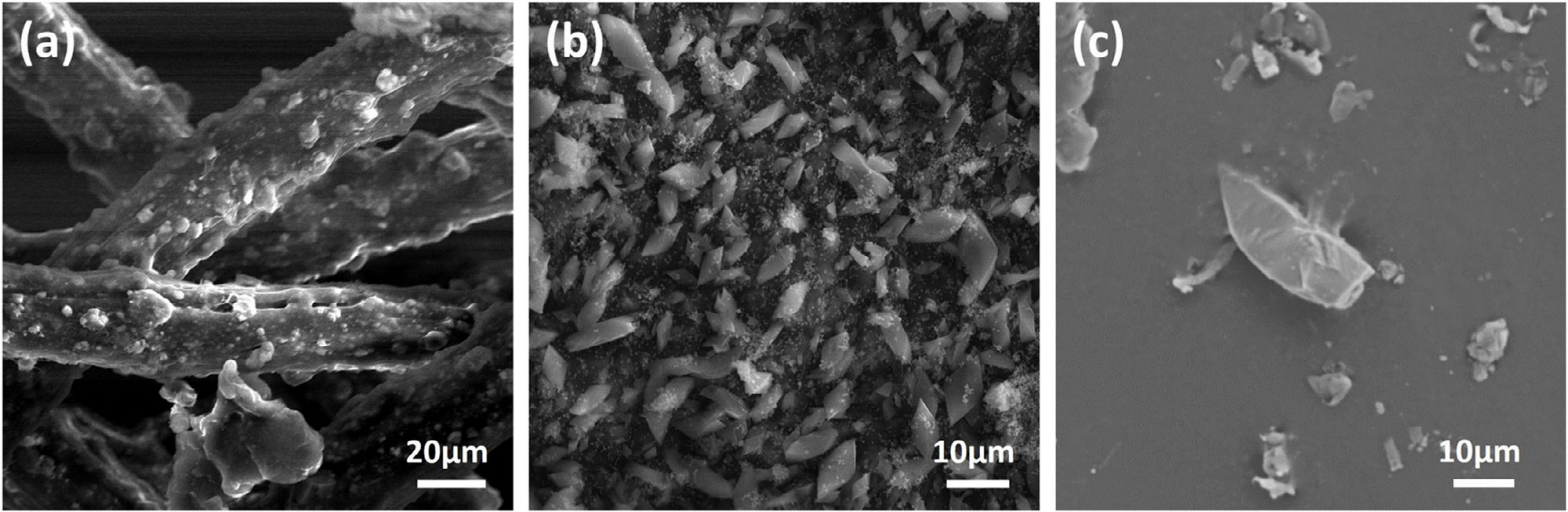
Morphological characterization by SEM. SEM images of (a) pristine PVDF‐UF membrane (MU_0_) without any MOF incorporation, (b) MU_3_ membrane demonstrating uniform distribution of hexagonal microspindle Fe‐MOF crystals over UF membrane strands, and (c) procured PET‐MP particles exhibiting irregular pellet‐like morphology with broad size variation.

SEM analysis of the bare and Fe‐MOF@UF membranes revealed the characteristic hexagonal microspindle geometry of NH_2_‐MIL‐101(Fe)‐MOF crystals [40], distributed across the membrane (Figure 2a,b). The extent of surface coverage increased with higher MOF loading (*n* = 1 to 4), as depicted in Figure S2, and further supported by the analysis of MOF dosage in Figure 4. These observations corroborate the earlier particle size distribution results, indicating a predominant distribution of MOF particles over the membrane surface. Similarly, the SEM images of the PET‐MP samples displayed mainly fibrous and pellet‐like morphologies (Figure 2c), with average dimensions consistent with the hydrodynamic size measurements.

**FIGURE 3:**
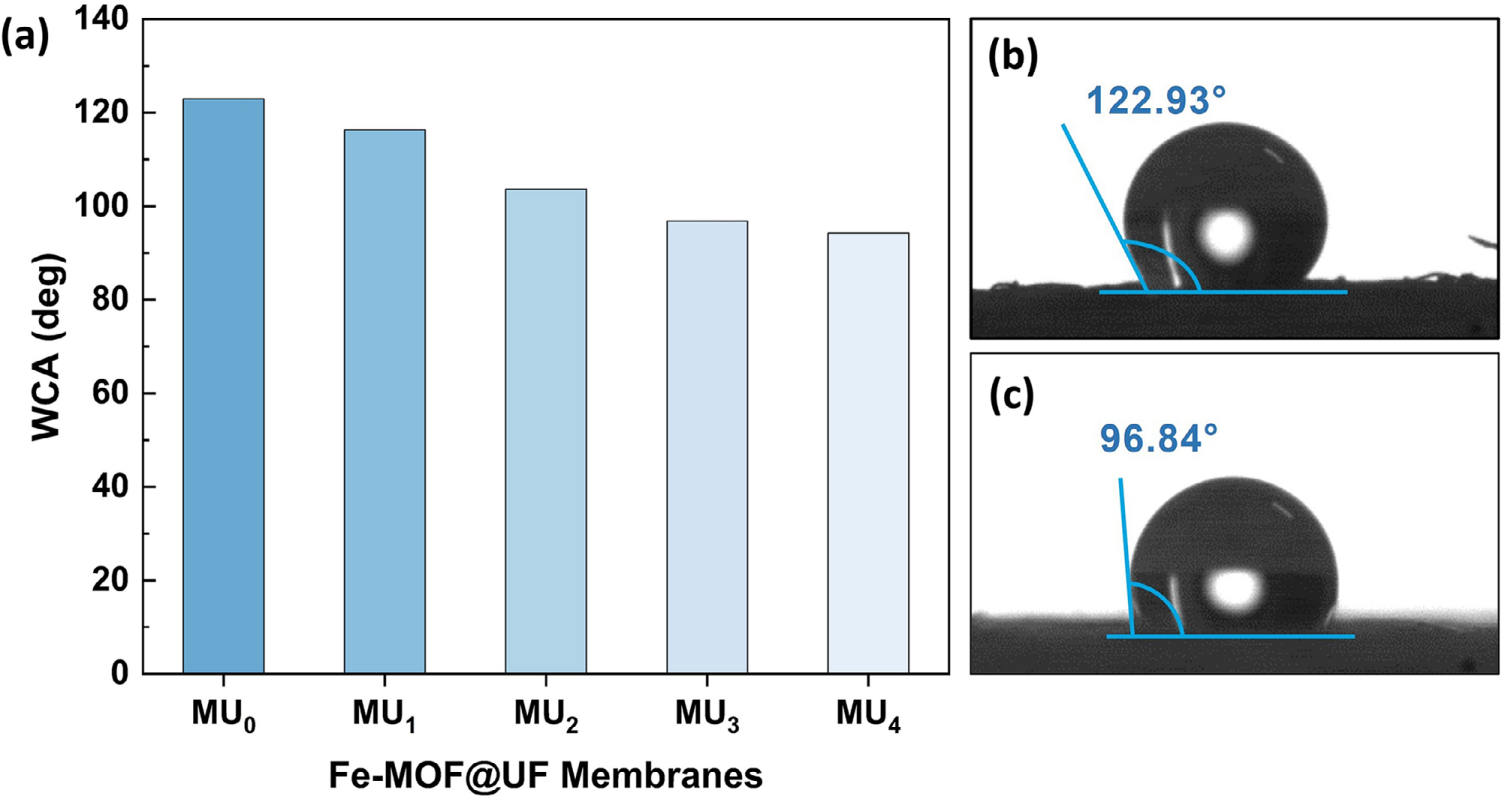
Water contact angle (WCA) evaluation. (a) The trend illustrates the variation of WCA from bare UF membrane (MU_0_) to synthesized Fe‐MOF@UF membranes (MU_1_ to MU_4_). Further, static WCA analysis images of (b) bare PVDF‐UF membrane (MU_0_), and (c) MU_3_ membrane, synthesized with a 3:3:1:500 precursor molar ratio, showcase the hydrophobicity of the respective membranes.

The membrane's hydrophobicity was assessed by measuring the water contact angle (WCA) using a contact angle goniometer. As shown in Figure 3a,b, the unmodified PVDF‐based UF membrane exhibited a high WCA, confirming its inherent hydrophobic nature relative to Fe‐MOF‐integrated membranes. This behavior is primarily attributed to the non‐polar α‐phase structure and the presence of ‐CF_2_‐ bonds that repel water [41]. However, Figure 3a illustrated a progressive decline in hydrophobicity, observed with increasing MOF dosage (from *n* = 1 to *n* = 4). This trend indicates the hydrophilic influence imparted by NH_2_‐MIL‐101(Fe)‐MOF dispersed over the membrane, whose surface‐functional amine and carboxyl groups foster strong polar interactions with water molecules, thereby enhancing surface wettability [42, 43]. Nevertheless, the composite membranes remained overall hydrophobic (WCA>90°) (Figure 3c), implying a need for pressure‐driven operation to facilitate water permeation.

### 2.2 Effect of MOF Dosage on PET‐MP Rejection

**FIGURE 4:**
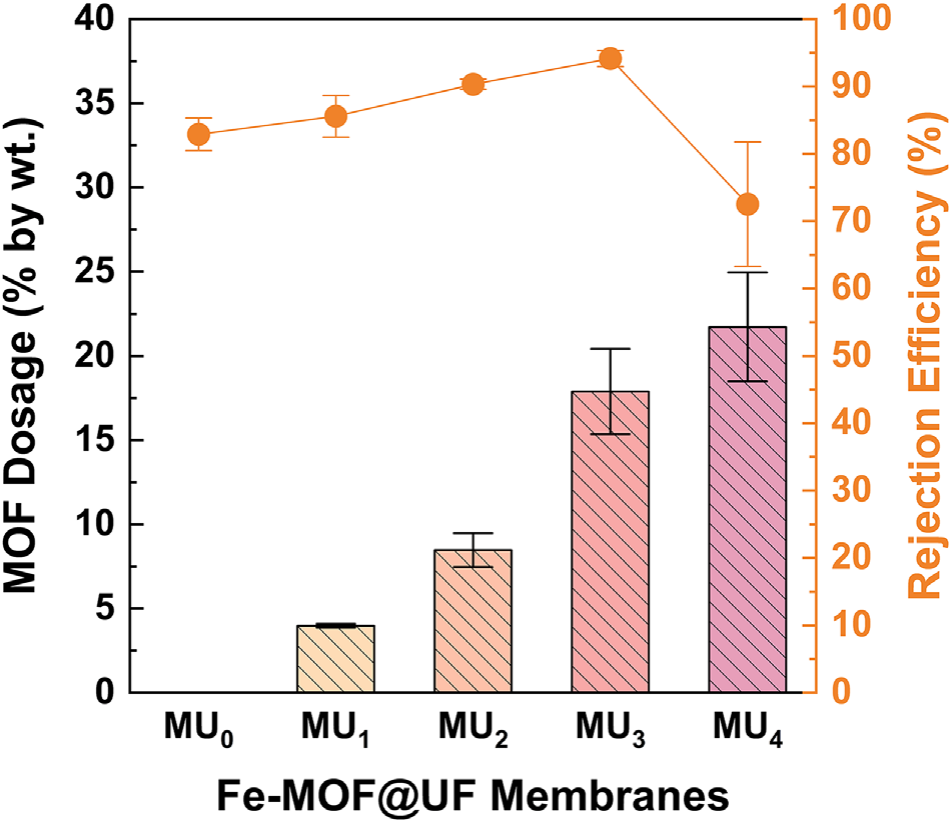
Influence of MOF dosage in PET‐MP rejection efficiencies. Column plots demonstrate the progressive increase in Fe‐MOF loading on PVDF‐UF membranes, from MU_1_ (molar ratio of 1:1:1:500) to MU_4_ (molar ratio of 4:4:1:500), along with the bare membrane (MU_0_) as the control. Additionally, closed orange circles represent corresponding PET‐MP removal efficiencies of each Fe‐MOF@UF membrane with varying MOF dosage. The error bars indicate the standard deviation (SD) at each measurement point.

In a solvothermal synthesis approach, the MOF loadings were systematically tuned within the composite membranes by varying the precursor concentrations [29]—specifically, Fe^3+^ salts and NH_2_‐BDC ligands in this study (Table S2). The initial synthesis of the MU_1_ membrane, fabricated with molar proportion of 1:1:1:500, yielded an average MOF loading of 4 ± 0.1%, which subsequently increased with higher precursor ratios—from 8.5 ± 0.5% in MU_2_ to 22 ± 2% in MU_4_ (Figure 4 and Table S2). This enhancement in MOF loading at higher metal‐ligand proportion is mainly ascribed to intensified complexation between the organic linkers and metal ions, which promotes more extensive nucleation and particle growth [44]. Ultimately, the increasing density of MOF particles adheres onto the available UF membrane surface, predominantly due to the hydrogen bonding interactions between the ‐CF_2_‐ units on the PVDF surface, and hydroxyl groups (OH–) of the MOF particles [29].

**FIGURE 5:**
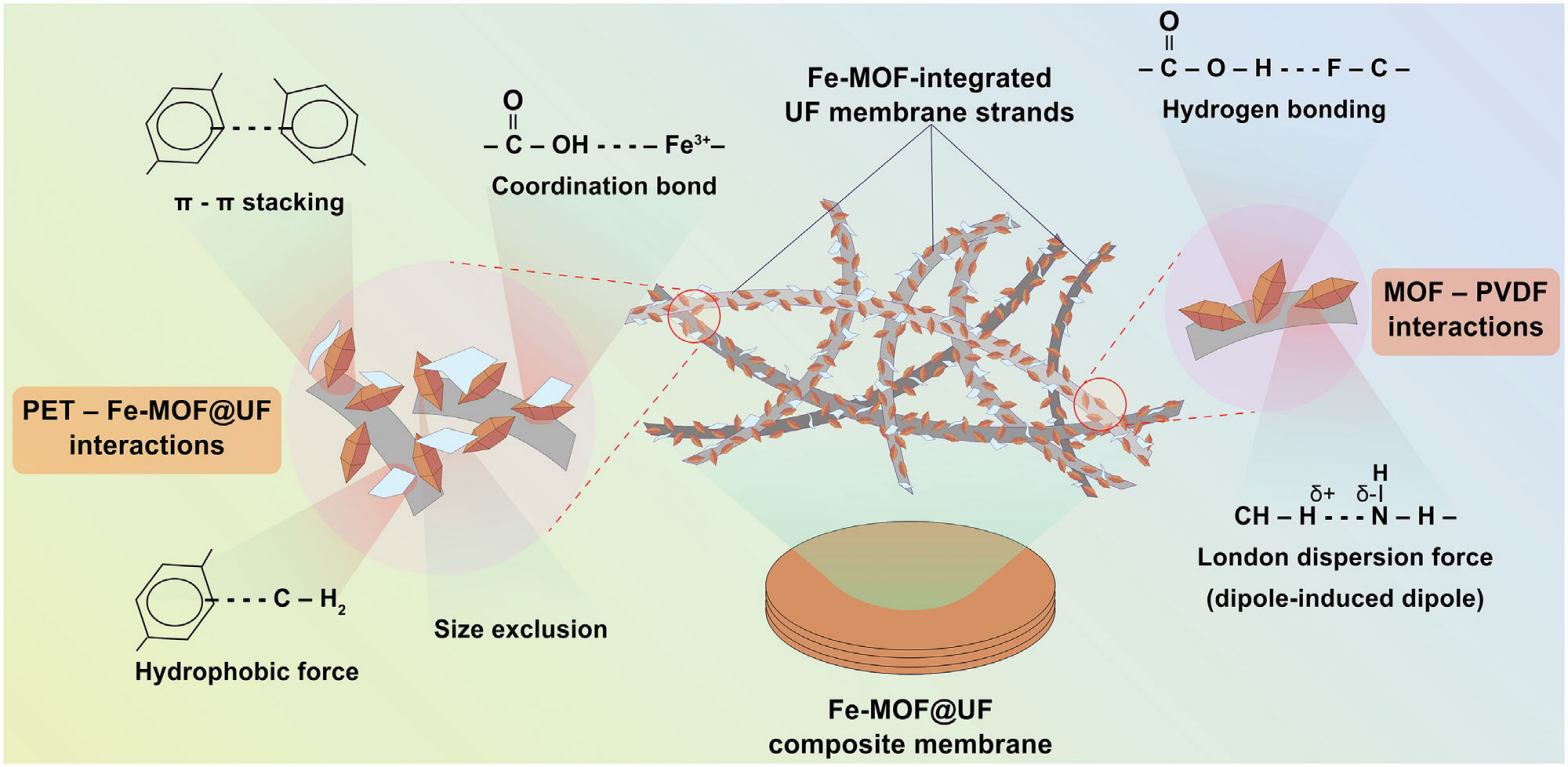
Interaction mechanisms. Schematic illustration shows the possible interaction mechanisms: Fe‐MOF particles with the PVDF‐UF membrane during the MOF‐integration phase **(right)**, and PET‐MPs with Fe‐MOF@UF membranes occurring during filtration phase **(left)**.

In succession, the PET‐MP removal efficacies of these membranes (MU_0_ to MU_4_) were investigated using a typical vacuum filtration setup (Figures S1 and S3). Here, the bare MU_0_ membrane served as a control, achieving a baseline removal efficiency of 83 ± 2% (Figure 4 and Table S2). Incorporation of NH_2_‐MIL‐101(Fe)‐MOF substantially improved the removal performance, peaking with the MU_3_ membrane at 94 ± 1%. This improvement is due to the increased MOF loading, which promotes elevated synergistic effects of size exclusion and strong interfacial interactions—including π–π stacking (between aromatic groups), coordination bonding (between MOF's metal centers and carboxylate groups on the PET surfaces), and hydrophobic interactions (between non‐polar groups of PVDF membrane and PET‐MPs) (Figure 5).

However, the MU_4_ membrane displayed a substantial decline in performance (73 ± 9%) despite the highest MOF loading (Figure 4 and Table S2), undoubtedly due to the MOF leaching from the membrane surface, as visually evidenced by the reddish‐brown discoloration of the filtrate (Figure S4a). Quantitative analysis further substantiated this observation, with the filtrate displaying an elevated total‐iron (T‐Fe) concentration of 1.4 ± 0.7 mg/l—measured via spectrophotometer under visible wavelength of ∼510 nm. This high iron content in the filtrate indicated substantial release of MOF‐derived constituents, especially iron species. This leaching is likely due to surface oversaturation, where excess MOF particles loosely stack on fully occupied membrane sites, forming weakly bound layers prone to detachment during filtration. Additionally, as Fe^3+^ ions are coordinated within the MOF framework, weak anchoring of MOF under oversaturated conditions may facilitate partial release of iron species into the filtrate, consistent with the observed high T‐Fe levels for the MU_4_ filtrate. Practically, such leaching of MOF particles into the filtered water could pose adverse health risks through the release of harmful organic and inorganic constituents, if consumed. Therefore, based on these outcomes, the MU_4_ membrane was excluded from further consideration, while the MU_3_ membrane—synthesized with a molar proportion of 3:3:1:500—was established as the optimally loaded configuration for effective PET‐MP removal and selected for subsequent analysis.

### 2.3 Membrane Performance Assessment

#### 2.3.1 Influence of Synthesis Parameters

**FIGURE 6:**
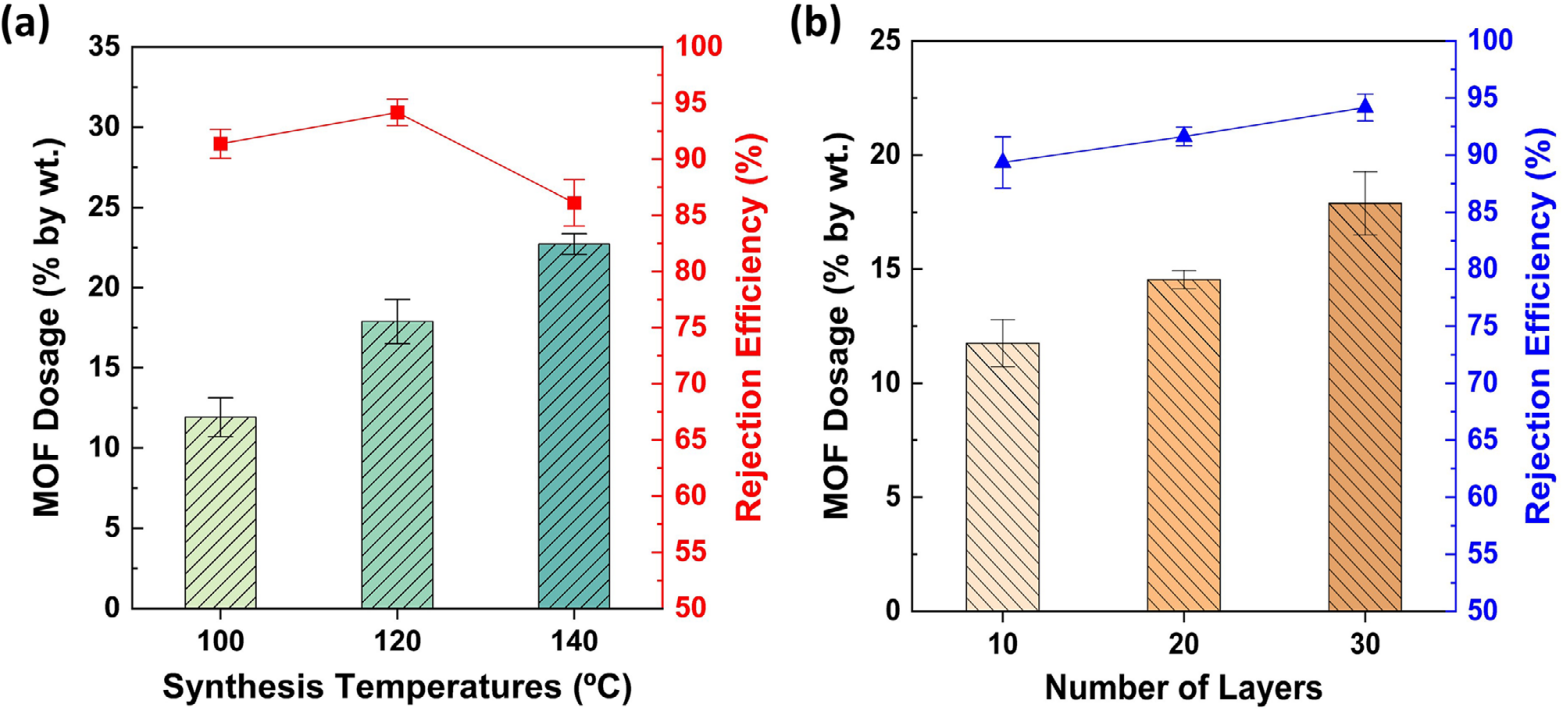
Variation of MOF loading and removal performance with respect to synthesis parameters. (a) Green bars demonstrate the variation in MOF dosage with increasing synthesis temperature for MU_3_ membranes (optimally synthesized at a molar ratio of 3:3:1:500), while closed red squares further illustrate the corresponding PET‐MP removal performance of MU_3_ membranes synthesized under each synthesis temperature. Likewise, (b) Orange columns depict the change in MOF loading with increasing numbers of membrane layers, and closed blue triangles show the corresponding variation of PET‐MP removal efficiencies of MU_3_ membranes fabricated with varying number of membrane layers. The error bars represent the standard deviations (SD) obtained during data curation.

To assess the influence of synthesis temperature on PET‐MP removal performance, optimally loaded MU_3_ membrane was synthesized at 100, 120, 140, and 160°C. The MOF loadings on the membranes synthesized at 100, 120, and 140°C are determined to be 12 ± 1%, 18 ± 2%, and 23 ± 1%, respectively (Figure 6a and Table S3). This progressive increase in MOF yield up to 140°C may indicate the temperature‐enhanced nucleation and crystallization kinetics, which promote greater crystal growth [45, 46, 47]. Subsequently, their PET‐MP rejection efficiencies follows a distinct trend, improving to a maximum at 120°C but declining at 140°C. The initial improvement can be attributed to the greater MOF deposition on the UF membrane resulting from increased synthesis temperature, which facilitates enhanced size exclusion and greater MOF‐MP interactions. However, for the membrane prepared at 140°C, despite high MOF yield, the limited surface area of the membrane likely led to oversaturation of MOF particles on the surface. This excessive surface loading may have resulted in loosely bound particle stacking, thereby promoting the subsequent leaching of Fe‐MOF into the filtrate (Figure S4b)—similar to that observed previously for MU_4_ membrane with reddish‐brown color. In the case of 160°C, the resulting system exhibited severe surface damage due to over‐burning (Figure S5) and was therefore excluded from further tests on MOF content and PET‐MP removal performance. Hence, 120°C is identified as the optimal synthesis temperature, accordingly, providing a balance between MOF yield and PET‐MP removal performance.

The PET‐MP removal performance of the MU_3_ membrane was evaluated by varying the number of membrane layers, aiming to optimize commercial filter design while maximizing efficiency following Fe‐MOF incorporation. Results revealed a clear upward trend of both MOF content and PET‐MP removal efficiency, with increasing layers (Figure 6b and Table S4). Here, MOF loading rose from 12 ± 1% for Fe‐MOF@UF membrane with 10 layers to 18 ± 1% for that with 30 layers, while PET‐MP rejection efficiency increased from 89 ± 2% to 94 ± 1%, respectively. This enhancement is primarily attributed to the increased availability of active sites for MOF particle adsorption on the membrane surface, which strengthens interfacial interactions with PET‐MPs and thereby improves overall removal performance due to the high MOF content. The observed trend further suggests that membranes with additional layers can enhance PET‐MP rejection in integrated filtration systems. Nonetheless, increasing the number of membrane layers risks exceeding the capacity of commercial membrane cartridges—such as the standard 4‐inch UF membrane cartridge—potentially compromising system compatibility and structural integrity. Therefore, future research should focus on strategies to achieve higher MOF loading within fewer membrane layers (i.e., fewer than 30), to maximize removal efficacy while investigating techno‐economic analysis for the commercialization of the membrane, maintaining compatibility with commercial cartridge dimensions, and ensuring practical feasibility for point‐of‐use (POU) filtration applications.

#### 2.3.2 Effect of Feed pH and Ionic Composition

**FIGURE 7:**
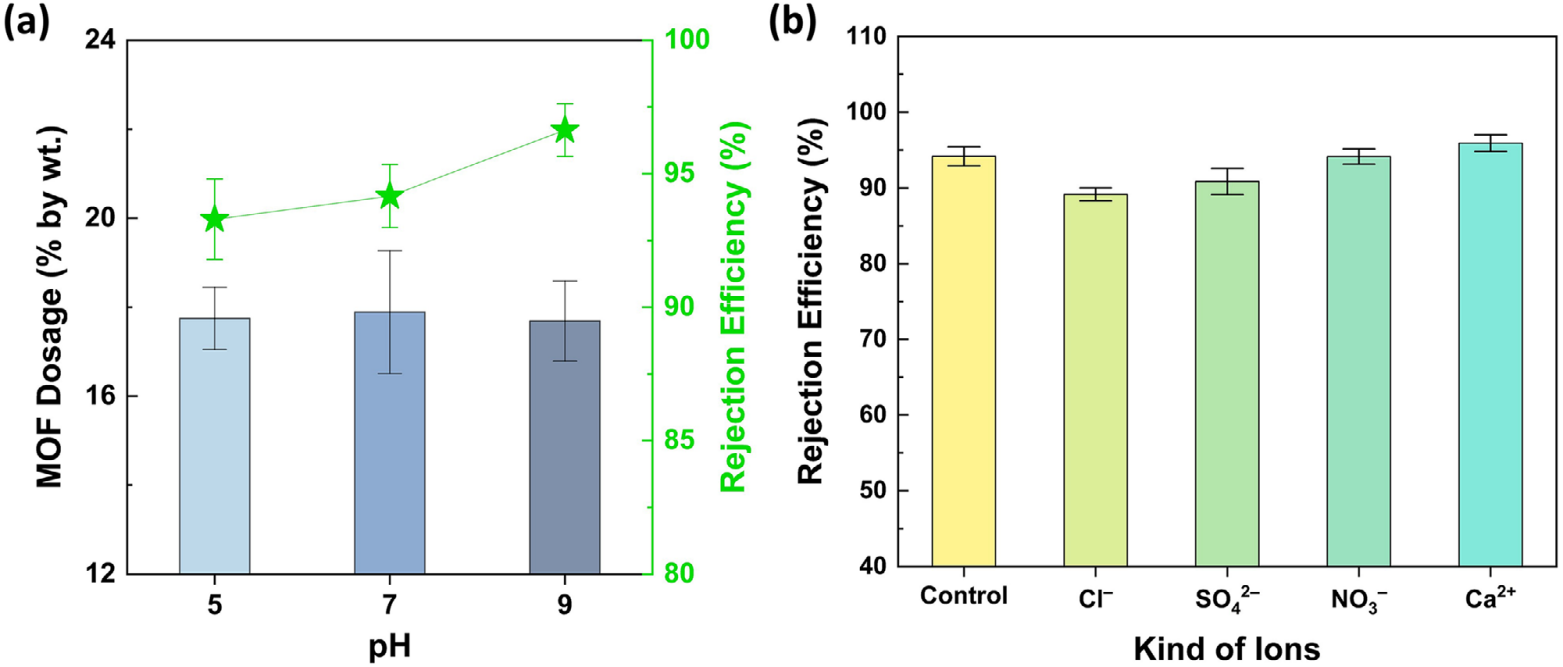
Influence of chemical parameters in PET‐MP removal performance. (a) Blue bars represent the variation in MOF loading on MU_3_ membrane, prepared under optimal synthesis conditions, with varying pH of the feed solution. Further, closed green stars indicate the corresponding change in PET‐MP removal efficiencies at each pH. Moreover, (b) column plots underscore the change in PET‐MP removal performance of the MU_3_ membrane due to the effects of different anions and cations existing in feed solution. The error bars demonstrate the standard deviations (SD) at corresponding measurements.

To assess the impact of feed pH on MP filtration performance, the PET‐MP rejection efficiencies of the MU_3_ membrane were evaluated at pH 5, 7, and 9—reflecting the standard drinking water pH range recommended by international guidelines [48, 49]. The results revealed the highest and lowest PET‐MP rejection efficiencies of 97 ± 1% and 93 ± 2%, respectively, recorded at pH 9 and 5 (Figure 7a and Table S5). These variations result from pH‐driven changes in the surface charge of both the adsorbent (Fe‐MOF dispersed on the membrane surface) and the adsorbate (PET‐MP suspended in feed solution) [39]. At higher pH, gradual deprotonation of the surface ammonium groups (–NH_3_^+^ to –NH_2_) on Fe‐MOF, and the dissociation of carboxylic acid moieties (–COOH to –COO–) on PET‐MPs increase the overall negative surface charge of both species (Figure 1d) [38, 39]. Although this mutual surface negativity could lead to electrostatic repulsion, this effect is likely outweighed by dominant size exclusion and specific adsorption mechanisms. These include strong coordination bonds formed between the exposed Fe^3^^+^ centers of the MOF and the pH‐induced coordination interactions with dissociated carboxylate (‐COO^−^) groups on PET‐MP functionalities. Additionally, these interactions are further reinforced by π–π stacking (between aromatic rings of MOF and PET‐MP particles) and hydrophobic forces (between hydrophobic PVDF membrane and PET‐MPs), collectively contributing to effective PET‐MP rejection under alkaline conditions. Therefore, the MU_3_ membrane consequently demonstrated superior PET‐MP removal efficiency at higher pH levels, indicating that slightly basic feeds are more optimal for operation. Notably, within the typical potable water pH range, the MOF‐integrated membrane successfully outperformed the unmodified UF membrane (MU_0_), underscoring the effectiveness of Fe‐MOF incorporation in minimizing MP exposure at POU.

Given that trace concentrations of inorganic ions are commonly present in drinking water and are maintained within guideline limits, it is essential to evaluate their influence on the membrane's MP removal performance. These ions can significantly affect the interactions between the adsorbent and the adsorbate, potentially altering adsorption dynamics and membrane efficiency. In this context, representative ions frequently found in potable water—Cl–, SO_4_^2–^, NO_3–_, and Ca^2+—^were introduced separately into the feed solution, and subsequently filtered through the MU_3_ membrane. The observed ionic interference on PET‐MP removal followed the order: Cl– > SO_4_^2–^ > NO_3–_ > Ca^2+^, aligning with results illustrated by previous studies [35, 50]. Specifically, PET‐MP rejection decreased from 94 ± 1% (control) to 89 ± 1%, 91 ± 2%, and 94 ± 1% in the presence of Cl–, SO_4_^2–^, and NO_3–_, respectively (Figure 7b and Table S6). This decline is likely due to these anions competing with the dissociated, negatively charged surface groups of PET‐MPs for active adsorption sites. In doing so, the anions compress the electric double layer (EDL) on the Fe‐MOF surface, substantially altering the particle surface charges. Consequently, charge‐driven interactions—such as coordination bonding and π‐π stacking between MOF particles and PET‐MPs, as inferred from FTIR analysis and MOF dosage‐dependent observations—are significantly weakened. Under these conditions, MP removal primarily relies on mechanical straining, and possible hydrophobic interactions only, thereby lowering overall removal efficiencies. In contrast, divalent Ca^2+^ ions enhanced PET‐MP removal to 96 ± 1% (Figure 7b and Table S6), due to their ability to bridge the negatively charged groups on both PET‐MPs and Fe‐MOF particles, thereby strengthening electrostatic attraction [51]. Overall, these findings demonstrate that the Fe‐MOF‐integrated MU_3_ membrane maintains high PET‐MP rejection efficiency, effectively functioning even in the presence of anions and cations in the feed solution, outperforming the bare MU_0_ membrane.

#### 2.3.3 Reusability of Fe‐MOF@UF Membranes

**FIGURE 8:**
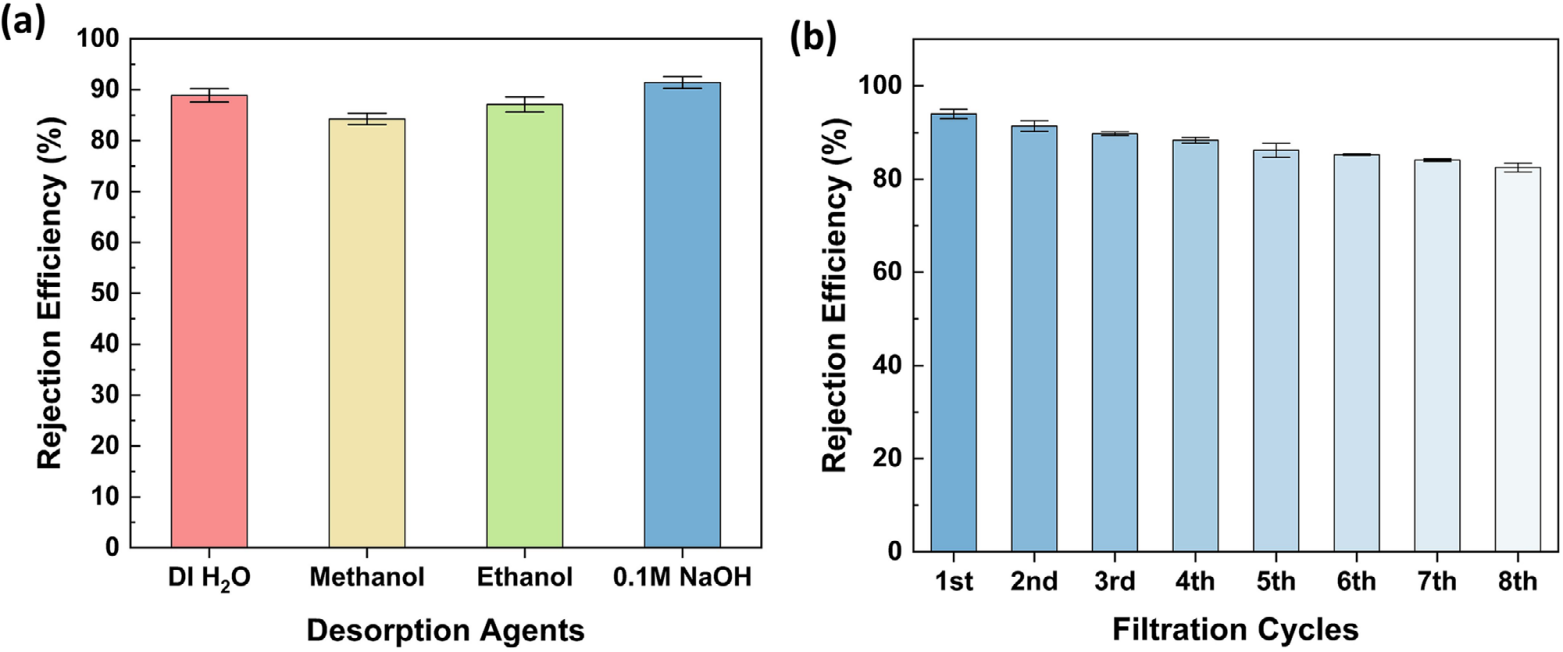
Assessment of reusability of the Fe‐MOF@UF membrane. (a) Distinct colored columns highlight the effect of various desorbing agents on the PET‐MP removal efficiency of the MU_3_ membrane during the second filtration cycle, following regeneration of the MOF‐integrated membrane with respective agents after first filtration. Subsequently, (b) progressive blue columns demonstrate the gradual decline in PET‐MP removal performance of the MU_3_ membrane over eight successive filtration cycles—with regeneration using 0.1 M NaOH (optimal desorbing agent).

Desorption studies were initially conducted using DI water (control), Methanol, Ethanol, and 0.1 M NaOH to identify an effective reagent for desorbing PET‐MPs retained on the Fe‐MOF@UF membrane following filtration [35]. Among them, 0.1 M NaOH demonstrated the best desorption performance, as reflected by the highest PET‐MP rejection efficiency of the membrane in the second filtration cycle after its use. Specifically, the MU_3_ membrane showed relatively minimal decline in efficiency, from 94 ± 1% in the first filtration cycle to 91 ± 1% in the subsequent cycle (Figure 8a and Table S7). The high desorption performance of NaOH is attributable to its strong alkalinity, which enhances the negative surface charge of both the adsorbent and adsorbate. This charge amplification intensifies electrostatic repulsion, effectively disrupting existing MOF‐MP interactions and thereby, removing PET‐MPs from the membrane surface. Therefore, 0.1 M NaOH was selected as the optimal desorbing agent for evaluating membrane reusability.

To comprehensively assess the membrane's reusability, the PET‐MP rejection performance of the MU_3_ membrane was systematically evaluated over multiple filtration‐desorption cycles until its efficiency approached that of the bare MU_0_ membrane (Table S8). Following each cycle, PET‐MPs retained on the membrane surface were desorbed using 0.1 M NaOH. As illustrated in Figure 8b and Table S8, the rejection efficiency progressively declined with successive cycles, a trend attributable to the gradual loss of Fe‐MOF particles during each desorption step, and corresponding loss of active adsorption sites within the Fe‐MOF@UF matrix [35]. Notably, despite this reduction, the Fe‐MOF@UF membrane retained over 83% efficiency even after eight successive adsorption‐desorption cycles, underscoring its robust reusability in a practical household drinking water purification system that facilitates the consumers at POU. Importantly, unlike the filtrate from the MU_4_ membrane (Figure S4a), no discoloration of the filtrate or detectable MOF leaching was observed in the filtrates from all eight cycles, suggesting the absence of MOF‐derived constituents at visually concerning risk levels. Additionally, as a representative mid‐cycle condition, filtrate from the fourth filtration cycle was subjected to partial quality analysis (Table S9 and Figure S6). The results further confirmed that no MOF‐related leaching had occurred that could adversely affect the chemical quality of the filtrate. Although this does not represent a comprehensive long‐term operational study, these findings assure the MOF stability within the optimized Fe‐MOF@UF membrane. Consequently, this demonstrates the membrane's ability to deliver water with no MOF leaching over consecutive filtration cycles, reinforcing its practical reusability for household POU water purification.

Although this performance of the Fe‐MOF@UF membrane system did not surpass other reported MOF‐polymer systems [28, 29], the findings of this study demonstrate that the optimally synthesized Fe‐MOF@UF membranes exhibit strong reusability. Moreover, these membranes support a straightforward and consumer‐friendly desorption process using NaOH solution, enabling efficient regeneration. These attributes highlight the practical potential of Fe‐MOF‐integrated membranes for POU applications, facilitating scalability in commercial, small‐scale water purification technologies.

### 2.4 Performance Validation

To investigate the practical feasibility of the synthesized Fe‐MOF@UF membrane system, performance validation was carried out using commercially available PET‐bottled drinking water samples. The initial MP concentration in the sampled drinking water is measured at 70 ± 12 particles L^−1^, which is reduced to 8 ± 8 after filtration through the optimized Fe‐MOF@UF membrane, corresponding to a removal efficiency of 89 ± 10% (Table S10). Although this value is marginally lower than that achieved by the controlled MU_3_ membrane, the decrease might be attributed to the presence of various coexisting ions and dissolved solutes in the bottled water, which likely hindered MP adsorption [35]. Nevertheless, the Fe‐MOF@UF membrane substantially outperformed the bare MU_0_ membrane, emphasizing its suitability for MP removal in real‐world POU scenarios. Additionally, while a quantitative assessment of the individual contributions of these coexisting constituents would provide deeper mechanistic insight, such analysis would require controlled water chemistry experiments which fall outside the current research's framework. Future investigations should systematically examine the effects of specific ionic compositions and concentrations to elucidate the MP removal mechanism under real‐world filtration scenarios.

Additionally, to investigate the potential the effectiveness of the Fe‐MOF@UF membrane, the filtrate was analyzed to confirm its potability. Different physicochemical parameters were tested and compared with different international drinking water quality guidelines [48, 49] (Table S11). The results confirmed that all the evaluated parameters of the filtrate remained within the permissible limits at the time of analysis, indicating that it would be safe for human consumption. This finding not only demonstrates the efficacy of Fe‐MOF@UF membranes to remove MPs at the POU, but also their capacity to deliver potable water that complies with established drinking water quality standards.

While the optimized Fe‐MOF@UF membrane exhibits slightly lower MP removal efficiency than some previously reported MOF‐integrated membrane systems (Table 1), this study fills a critical gap by demonstrating the direct application of MOF‐membrane composites in PET‐MP removal for drinking water purification—an area not previously explored. The inclusion of wastewater and seawater studies in Table 1 provides a performance benchmark, underscoring the Fe‐MOF@UF membrane's compatibility with existing MOF‐membrane technologies, particularly in the drinking water domain, while highlighting its potential for further optimization. Unlike most prior studies limited to lab‐scale synthesis, this work innovatively integrates NH_2_‐MIL‐101(Fe)‐MOF onto a commercially available UF membrane commonly used in household filtration, offering a realistic, feasible, and scalable pathway for real‐world POU applications for enhanced MP removal. Therefore, despite its relatively modest rejection performance, this study marks a critical step toward scalable implementation of Fe‐MOF@UF membranes, highlighting their potential as reliable candidates for household MP removal from drinking water at the POU, while delivering safe and potable drinking water quality in line with international standards.

**TABLE 1:** Comparative assessment of the Fe‐MOF@UF membrane against reported MOF‐integrated membrane systems for MP separation performance.

**MOF‐integrated membrane name**	**Integrated MOF species**	**Substrate system**	**Polymer removed**	**Simulated feed condition**	**MP rejection efficiency [%]**	**Refs**.
TFC FO	MIL‐53 (Fe)	PSF substrate	PE ^a^	Wastewater	Not mentioned	[27]
PSF/MIL‐100 (Fe)	MIL‐100 (Fe)	PSF matrix	PVC ^b^ and PE	Wastewater	99.9%	[28]
UiO‐66‐OH@MF‐3	UiO‐66‐OH	Melamine foam	PVDF ^c^, PS ^d^, and PMMA ^e^	Seawater	95.5%	[29]
Fe‐MOF@UF	NH_2_‐MIL‐101 (Fe)	Commercial PVDF UF membrane	PET	Drinking water	94.2 ± 1.2%	This work

^a^ Polyethylene (PE);

^b^ Polyvinyl Chloride (PVC);

^c^ Polyvinylidene Fluoride (PVDF);

^d^ Polystyrene (PS);

^e^ Polymethyl methacrylate (PMMA).

## 3 Conclusions

Microplastics (MPs) are increasingly recognized as contaminants of concern, and their presence in commercially available PET‐bottled drinking water has been widely reported. With growing reliance on bottled water and the potential for MPs to carry other harmful substances, reducing exposure to, particularly, PET‐based MPs at the point‐of‐use (POU) is a growing challenge. In this study, a novel Fe‐MOF@UF membrane was developed by incorporating NH_2_‐MIL‐101(Fe)‐MOF onto a commercial PVDF‐based ultrafiltration (UF) membrane using a solvothermal method. Under optimized conditions, the membrane achieved a PET‐MP rejection efficiency of 94 ± 1%, driven by strong physicochemical interactions between the MOF particles and the MPs. The membrane's performance was tested and optimized under various synthesis and operational conditions, whereas its ability to be reused using a simple 0.1 M NaOH wash further supports its practicality. When applied to real PET‐bottled water samples, the membrane removed approximately 89% of MPs, while delivering potable water meeting international drinking water standards. These results point to a promising solution for MP removal in everyday drinking water purification.

Looking ahead, future research should prioritize detailed evaluation of advanced membrane characteristics and operational parameters, including membrane durability, pore structure, binding site density, water flux, and antifouling properties, to fully assess the broader practical applicability of Fe‐MOF@UF membranes. The performance of these MOF‐based membranes should also be investigated against a wide range of MP polymers to reflect real‐world purification. Furthermore, comprehensive techno‐economic assessments are essential to evaluate the commercialization potential and development of cost‐effective MOF‐membrane systems. While such assessments were beyond the defined scope of the present study and limited by the unavailability of specialized analytical resources, this work carefully establishes a foundational proof‐of‐concept for the development of MOF‐UF composite filtration technologies, demonstrating effective MP removal and potable water delivery, thereby supporting safe, scalable, and sustainable POU systems for safe drinking water.

## Author Contributions

**Sahil Shrestha**: Conceptualization, Methodology, Formal analysis, Investigation, Data curation, Software, Visualization, Writing – original draft. **Ajaya Subedi**: Methodology, Visualization, Writing – review and editing. **Shane A. Snyder**: Investigation, Validation, Writing – review and editing. **Michael J. Angove**: Methodology, Validation, Writing – review and editing. **Shukra Raj Paudel**: Conceptualization, Investigation, Validation, Resources, Supervision, Project administration, Writing – review and editing.

## Conflicts of Interest

The authors declare no conflicts of interest.

## Supporting information




**Supporting File**: gch270089‐sup‐0001‐SuppMat.docx

## Data Availability

The data that support the findings of this study are openly available in Mendeley Data at https://doi.org/10.17632/j3mzhnxfdc.1, reference number 0.
