# Supplementary material for: Toward Scalability: Fe‐MOF‐Based Ultrafiltration Membrane for Effective Microplastics Removal from Drinking Water at Point‐of‐Use
Source: Glob Chall. 2026 Jan 7;10(1):e00559. doi: 10.1002/gch2.202500559 (PMC12780347; doi:10.1002/gch2.202500559)
Supplement: Supplementary file 1 — Supporting File: gch270089‐sup‐0001‐SuppMat.docx [file GCH2-10-e00559-s001.docx]

Supporting Information

**Towards Scalability: Fe-MOF-based Ultrafiltration Membrane for Effective Microplastics Removal from Drinking Water at Point-of-Use**

Sahil Shrestha**[
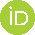
](https://orcid.org/0009-0001-3624-3130)**, Ajaya Subedi**[
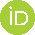
](https://orcid.org/0009-0008-3691-250X)**, Shane A. Snyder**[
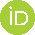
](https://orcid.org/0000-0003-2709-9840)**, Michael J. Angove**[
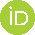
](https://orcid.org/0000-0002-0305-5168)**, Shukra Raj Paudel**[
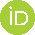
](https://orcid.org/0000-0002-5764-2990)***

**Part S1. Materials and Instrumentation**

*Materials*

All chemicals and reagents utilized in this study were of analytical grade and employed without any prior treatment. Specifically, 2-Aminoterephthalic acid (NH_2_-BDC, 98%) was sourced from BLD Pharmatech Pvt. Ltd. (Telangana, India), while Ferric (III) Chloride Hexahydrate (FeCl3.6H2O, 97%) and N, N-Dimethylformamide (DMF, 99.5%) were acquired from Sisco Research Laboratories (Mumbai, India). Deionized (DI) water was consistently prepared in-house through distillation followed by ion exchange. Additional materials, including commercial household UF membrane filter cartridges (4-inch long; pore size 0.1-0.01 µm) and Polyethylene Terephthalate particles (PET; < 1 mm; Mw ≈ 2.5 × 104 gm/mol), were purchased from SKC International Traders Pvt. Ltd. (Kathmandu, Nepal) and Baluna Industries Pvt. Ltd. (Kathmandu, Nepal), respectively. Other chemicals required for the experiments were obtained from various commercial suppliers.

*Characterizations*

The structural morphology of the synthesized materials was examined using a Scanning Electron Microscope (SEM, JEOL JSM-IT100). Functional groups of relevant materials were characterized via a Fourier Transform Infrared spectrometer (FT-IR, PerkinElmer Spectrum Two L160000A) in attenuated total reflectance (ATR) mode, within the spectral range of 4000 – 400 cm-1. Crystalline properties were analyzed using X-ray Diffractometer (XRD, Bruker D2 phaser) over a 2θ scan range of 8 - 40°. Particle size distribution and zeta (ζ) potential of PET-MPs and synthesized MOF were determined using a Dynamic Light Scattering analyzer (DLS, Horiba Scientific Nano Particle Analyzer SZ-100V2). Additionally, membrane surface hydrophobicity was assessed via a contact angle goniometer (Goniometer, Ramé-Hart Model 200-F1).

**Part S2. Experimental Procedures**

*MOF Synthesis and Membrane Fabrication*

In this study, Fe-MOF was synthesized and concurrently integrated onto a UF membrane using a solvothermal approach adapted from previously reported protocols,^[1–3]^ with substantial procedural modifications. Specifically, a solution was prepared by adding FeCl_3_.6H_2_O, NH_2_-BDC, and DI water in 50 mL DMF, maintaining a molar ratio of 1:1:1:500 (Fe^3+^:NH_2_-BDC:H_2_O:DMF) under continuous stirring (**Figure S1** and **Figure S3a**). In parallel, the procured commercial UF membrane filter cartridge was carefully disassembled. However, efforts to reassemble this cylindrical UF membrane into its original configuration within the cartridge proved challenging due to difficulties in achieving proper placement and an airtight seal. To overcome this, the membrane layers were unwrapped and cut into roughly circular discs (diameter ~50mm), with the number of sectioned units corresponding to the original number of membrane layers (N ~ 30) rolled around the cylindrical core. Then, these units were vertically stacked to form a stratified membrane assembly—designed to simulate the perpendicular flow regime as observed in practical filtration systems (**Figure S3b**).

The membrane stack and precursor solution were transferred to a Teflon-lined stainless-steel autoclave (Figure S1). The contents were subjected to ultrasonication for 15 minutes to promote uniform particle dispersion, followed by solvothermal treatment at 120°C for 48 hours. Upon cooling to room temperature, a reddish-brown Fe-MOF layer was visibly deposited on the membrane surface (**Figure S3c**). The resulting MOF-UF composite was rinsed thrice with DMF to remove loosely bound MOF particulates. Subsequent drying of the system at 60°C overnight yielded the final Fe-MOF-integrated UF membrane system. This Fe-MOF-modified membrane system was represented as ‘Fe-MOF@UF’ throughout the study, and was subjected to further analysis and characterization.

Initially, the Fe-MOF@UF composite membrane was characteristically designated as ‘MU_1_’, where the suffix ‘1’ denotes the 1:1 proportion of Fe^3+^ and NH_2_-BDC employed in the mix during synthesis. In subsequent experiments, this molar proportion was systematically varied—by elevating metal-to-ligand proportions to 2:2, 3:3, and 4:4—and the corresponding membranes were denoted accordingly (**Table S1**), to further investigate the influence of MOF loading on MP removal efficiency.


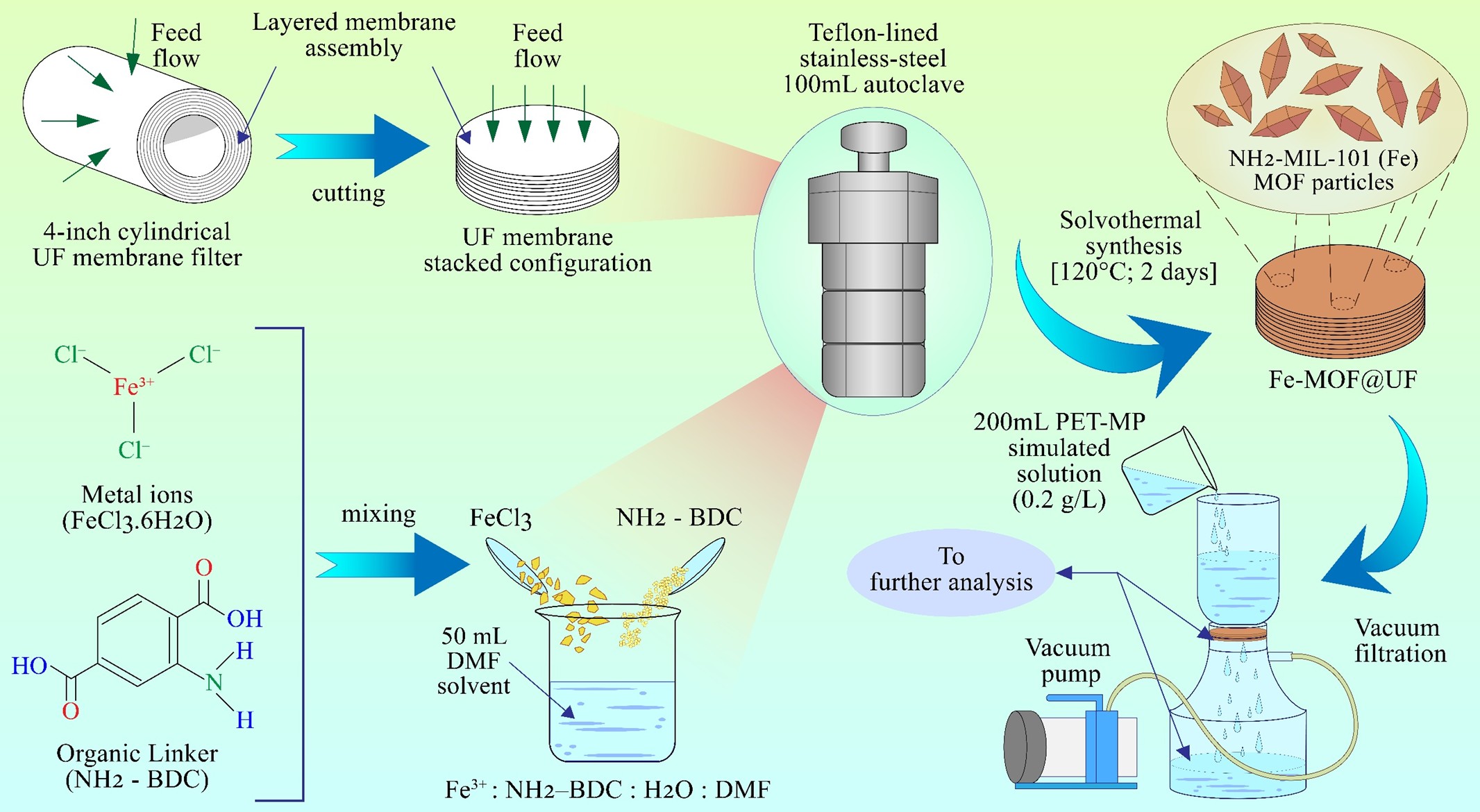


**Figure S1. Experimental design.** Schematic representation underscores the overall experimental workflow for synthesizing the composite MOF-integrated (Fe-MOF@UF) membrane. Furthermore, the figure depicts the experimental setup comprising of the laboratory vacuum filtration apparatus used for PET-MP rejection analysis, with Fe-MOF@UF membrane installed as filters for pressure-driven filtration.

*Experimental Protocol and MP Rejection Efficiency*

The present study evaluates the performance of the Fe-MOF@UF membrane against simulated water containing PET-MPs, to comprehensively investigate its PET-MP removal efficacy from drinking water at the POU in a sustainable and potentially scalable manner. Accordingly, a 200 mL DI water sample was prepared with an initial PET-MP concentration of approximately 0.2 g/L, followed by 15 minutes of ultrasonication for uniform dispersion of MP particles in the solution. For the filtration process under a controlled laboratory framework, a vacuum filtration apparatus was utilized with the synthesized Fe-MOF@UF membrane placed on the filter holder (Figure S1 and **Figure S3d**). Although vacuum-driven filtration differs from pressure-driven filtration (common in household units) in the mode of force applied, water transport in both is governed by transmembrane pressure, while the fundamental separation mechanism remains unchanged. Moreover, as a commercial UF membrane—also commonly used in household filtration units—was employed as a substrate throughout this study, this ensured direct material relevance to real-world applications. Thus, the vacuum filtration setup offered a reliable framework for evaluating membrane performance comparable to conventional household filtration conditions, whereas validation with complete household filtration units is recognized as an important avenue for future research.

For the MP suspension, PET-MPs were intentionally selected as the primary model contaminant, as commercial bottled waters—particularly those packaged in single-use or reused PET bottles—are widely reported to contain PET-based MPs.^[4, 5]^ While actual drinking water may contain a broader range of MP polymers, PET-MPs constitute the dominant fraction, making them a relevant target for evaluating membrane removal performance. Accordingly, a PET-MP suspension solution (~0.2 g/L) was then vacuum-filtered through the membrane, and the resulting filtrate was collected for further analysis. The PET-MP rejection efficiency of the Fe-MOF@UF membrane was evaluated based on the methodology outlined in previous literature.^[1]^ Initially, the initial and final concentrations of PET-MPs in the feed and filtrate, respectively, were determined gravimetrically. For which, the dry weight of a clean 50 mL beaker was recorded as ‘m_A_’ (g). Then, 20 mL of the filtrate was transferred into this beaker, and the beaker was dried in a convective oven at 140°C for 6 hours. After cooling, the beaker’s weight with retained MP residues was quantified as ‘m_B_’ (g). The initial concentration (C_i_) and final concentration (C_f_) (g/L) was calculated using **Equation** **S1**.

| $\text{C}_{\text{i/f}}\text{ =} \frac{\text{m}_{\text{B}} - \text{m}_{\text{A}}}{\text{V}} \text{× 1000}$ | (S1) |
| --- | --- |

Where V is the volume of the filtrate used (in mL).

Subsequently, the rejection efficiency (RE%) of the Fe-MOF@UF membrane system was achieved using **Equation** **S2**.

| $\text{RE\% =} \frac{\text{C}_{\text{i}} - \text{C}_{\text{f}}}{\text{C}_{\text{i}}}\text{ × 100\%}$ | (S2) |
| --- | --- |

where, C_i_ (g/L) and C_f_ (g/L) represent the initial and final concentrations of PET-MPs in the simulated feed sample and filtrate solution, respectively.

All the filtration tests were conducted in triplicates using independent samples, and the results were reported as mean values with corresponding standard deviations.

*Membrane Performance Assessment*

The performance of the Fe-MOF@UF composite membrane was systematically evaluated under various operational parameters—including synthesis temperature, MOF dosage, feed solution pH, ionic composition, number of membrane layers in the filtration setup, and choice of desorbing agent relevant to membrane reusability—by adopting the procedures outlined below:

Effect of MOF dosage: To investigate the effect of MOF dosage on MP removal performance of MOF-UF membranes, the molar concentrations of FeCl_3_.6H_2_O and NH_2_-BDC were systematically varied to achieve different MOF loadings (% by weight) on UF membranes.^[1]^ Initially, the commercial UF membrane was reconfigured (Figure S1 and Figure S3a), and its dry weight (m_1_) was recorded. The membrane was then subjected to baseline synthesis—under optimal synthesis temperature—using a 1:1:1:500 molar ratio of FeCl_3_.6H_2_O, NH_2_-BDC, DI water, and DMF ^[2]^, to fabricate a composite membrane system abbreviated as ‘MU_1_’ (Table S1). After MOF integration and subsequent overnight drying, the final weight (m_2_) of the membrane was measured, and the corresponding MOF loading was calculated using **Equation** **S3**.

| $\text{\% MOF dosage = }\frac{\text{m}_{\text{2}}-\text{m}_{\text{1}}}{\text{m}_{\text{1}}} \text{× 100\%}$ | (S3) |
| --- | --- |

Further, the PET-MP removal efficiency of Fe-MOF@UF-1 was determined utilizing Equation S1 and S2.

Membranes with higher MOF loadings were prepared by proportionally scaling the molar quantities of FeCl_3_.6H_2_O and NH_2_-BDC to 2:2, 3:3, and 4:4, respectively, while maintaining the amounts of DI water and DMF constant (Table S1). Each resulting MOF-integrated membrane, abbreviated as MU_2_ to MU_4_, underwent the same weight-based calculation and PET-MP removal testing. In addition, the PET-MP removal efficiency of a bare UF membrane, without any MOF modification, was also measured under identical conditions. This unmodified membrane served as a control to benchmark the performance of the composite membranes.

Moreover, to assess potential leaching of MOF-derived components, if required, into any filtrate after filtration through the synthesized membranes, the total iron (T-Fe) concentrations in the filtrate were quantitatively measured using a spectrophotometer (ASV11D, AS ONE Corporation, Japan) at visible wavelengths of ~510 nm. Monitoring these T-Fe levels allowed an approximation of whether MOF-related contents were released into the filtrate at levels of potential concern. Consequently, this guided the selection of the optimally loaded MOF-membrane system in conjunction with the resulting MP removal performance for subsequent analysis.

Finally, the MOF dosage showcasing the highest removal efficiency, together with no leaching of MOF-based contents, was selected for further analysis, and its associated MOF content was identified as the optimal dosage for UF membrane functionalization.

Effect of synthesis temperature: The influence of synthesis temperature on the MP removal performance of Fe-MOF@UF membranes was evaluated by conducting solvothermal synthesis at 100°C, 120°C, 140°C and 160°C. Following synthesis, membranes were visually examined, and those exhibiting signs of over-burning were discarded from further analysis. Only those with uniform reddish-brown coloration, smooth surface texture, and consistent MOF deposition were subjected to performance testing via filtration. The PET-MP concentrations in the filtrate and removal efficiencies of the Fe-MOF@UF were calculated using Equation S1 and S2, respectively. The synthesis temperature resulting in relative maximum PET-MP removal performance was indicated as the optimum temperature for Fe-MOF@UF membrane system.

Effect of membrane stratification: Initially, the stacked membrane configuration replicated the number of layers present in the original cylindrical UF membrane. However, it is hypothesized that integrating MOFs could enhance MP removal efficiency with even fewer layers—thereby reducing membrane material while supporting cost-efficient and scalable performance of this composite membrane in real-world applications. To evaluate this, the disassembled UF membranes were consequently segmented into stacks of 10, 20, and 30 layers. MOF-functionalized UF membranes were synthesized under optimized synthesis conditions, and their PET-MP removal efficiencies were determined using Equation S2. Ultimately, the configuration with the highest efficiency was selected for subsequent analysis.

Effect of pH: According to the World Health Organization and the United Nations Environmental Protection Agency,^[6, 7]^ the acceptable pH range of drinking water lies between 6.5 and 8.5. Following this, the pH of MP-simulated feed solutions was adjusted to 5, 7, and 9, to investigate the influence of pH on the removal performance of the Fe-MOF@UF membrane. Since the PET-MP suspension was initially prepared in DI water, the baseline pH of the feed solution was approximately 7.0, as recorded by a calibrated pH meter (HI2020-01, Hanna instruments). This unadjusted solution was used to assess the membrane performance at neutral pH. Subsequently, the pH the feed solution was adjusted by gradually adding HCl and NaOH solutions, while monitoring the pH until the desired pH was achieved. Once adjusted, the feed solutions were filtered through Fe-MOF@UF membranes that were synthesized under optimized conditions. Finally, the PET-MP concentration in the filtrate was determined, and the membrane’s rejection efficiency was evaluated to assess the influence of feed solution pH on the membrane’s MP removal performance.

Effect of ionic composition: The presence of various ions in the feed solution during filtration can affect the PET-MP removal performance of the Fe-MOF@UF membrane. To evaluate this effect, commonly occurring ions in drinking water—including Cl^-^, SO_4_^-2^, NO_3_^-^, and Ca^2+^—were introduced into 200 mL feed solutions at concentrations of 100 mg/L,^[3]^ using high-purity laboratory-grade chemicals. The solutions were thoroughly stirred and filtered through Fe-MOF@UF membranes under optimized operating conditions. The final PET-MP concentration in the filtrate was then measured using Equation S1, and the membrane’s rejection efficiency was assessed, using Equation S2, to determine the impact of feed’s ionic composition on the PET-MP removal performance.

Reusability of Fe-MOF@UF membrane: To assess the reusability of the Fe-MOF@UF membrane, a suitable desorbing agent was first identified based on its ability to restore high PET-MP removal efficiency in subsequent filtration cycles. For this, an initial filtration was performed under optimized operating conditions, and the membrane’s MP removal efficiency was measured as a baseline, following the previously mentioned procedures. The membrane was then washed three times with various desorbing agents—including DI water (control), Methanol, Ethanol, and 0.1M NaOH^[3]^—to remove retained PET-MP residue. Specifically, these hydroxide-containing reagents are utilized as desorbing agents,^[8]^ as they raise the pH of the desorbing medium, dissociate carboxyl functional groups, and disrupt existing coordination bonds. This enhances electrostatic repulsion between Fe-MOF and PET-MP particles, thereby effectively removes adhered PET-MPs from the Fe-MOF@UF membrane surface. Consequently, the membrane was dried at 60°C for 2 hours and reused for a 2^nd^ filtration cycle. The desorbing agent that achieved the highest recovery of removal efficiency was selected for the reusability study, as highest removal efficiency indicates higher regeneration of Fe-MOF particles over the membrane surface.

With the optimal desorbing agent, the Fe-MOF@UF membrane was subjected to multiple filtration cycles. After each cycle, the membrane was washed and dried with this desorbing agent followed by DI water, and its PET-MP removal efficiency was recorded adopting the same protocol. This process continued until the removal efficiency declined to a minimum threshold—presumably corresponding to the efficiency of the bare membrane under the control condition. The total number of effective filtration cycles completed before this performance drop was designated as the membrane’s maximum reusability limit.

*MP extraction and membrane performance validation*

Fe-MOF@UF membranes were ultimately fabricated, using optimized synthesis and operational parameters, including MOF dosage, synthesis temperature, and membrane layering, and was validated using real-world samples of commercially available PET-bottled drinking water. In this regard, MP pretreatment and extraction were conducted in accordance with the National Oceanic and Atmospheric Administration (NOAA) guideline,^[9]^ with slight adjustments adapted to suit the characteristics of bottled water samples.

From each acquired sample, 200 mL was allocated for pretreatment, along with 200 mL of DI water as a procedural blank to detect any potential MP contamination during sample processing. Each subsample—including bottled water and blanks—was treated with 20 mL of 30% hydrogen peroxide (H_2_O_2_) and 20 mL of aqueous 0.05M Fe (II) solution to eliminate any microbial matter while preserving MP integrity. These mixtures were heated at 70°C for 30 minutes (uncovered), facilitating oxidative digestion. Following this, the beaker then left at room temperature for 24 hours by covering with aluminum foil, to allow further digestion and passive sedimentation. Since the samples lacked visible sediment content, the standard density separation with NaCl was not performed.

Post-digestion, the supernatant was carefully vacuum-filtered through 0.45µm cellulose nitrate filter paper (47mm diameter, 3.1mm grid). Then, the filters were air-dried in sterile Petri dishes, and examined under a binocular compound microscope (Olympus CX22RFS1) at 40X-100X magnification range. MPs larger than ~10µm were counted, and the mean initial MP concentrations in bottled-water samples were recorded.

After filtration through Fe-MOF@UF, the final MP concentration in the filtrate was evaluated using the same digestion and microscopic analysis protocol, and the MP removal efficiency of Fe-MOF@UF for actual commercial drinking water purification was determined to validate its performance in real-world application and promote its scalability. Ultimately, key water quality parameters of the filtrate— such as pH, conductivity, total dissolved solids (TDS), turbidity, hardness, chloride, iron, arsenic, and other contents—were investigated to evaluate the potability of water in compliance with international drinking water quality standards, and consequently, support the viability of the Fe-MOF@UF membrane for practical drinking water purification applications.

Additionally, the primary aim of this study was to synthesize and optimize the Fe-MOF@UF composite membrane and assess its efficiency in removing MPs from drinking water, with particular emphasis on its ability to produce potable water for scalability. As such, advanced membrane characteristics and mechanistic performance parameters—such as pore volume, pure water flux, surface area, filtration rate, and antifouling properties—were not measured as their evaluation and validation required experimental procedures and specialized resources exceeding the intended focus of this work. Nevertheless, this work establishes a solid foundation for future optimization of MOF-membrane composites, including Fe-MOF@UF membranes, for scalable, sustainable, and effective POU drinking water purification.

**Part S3. Characterization Results**


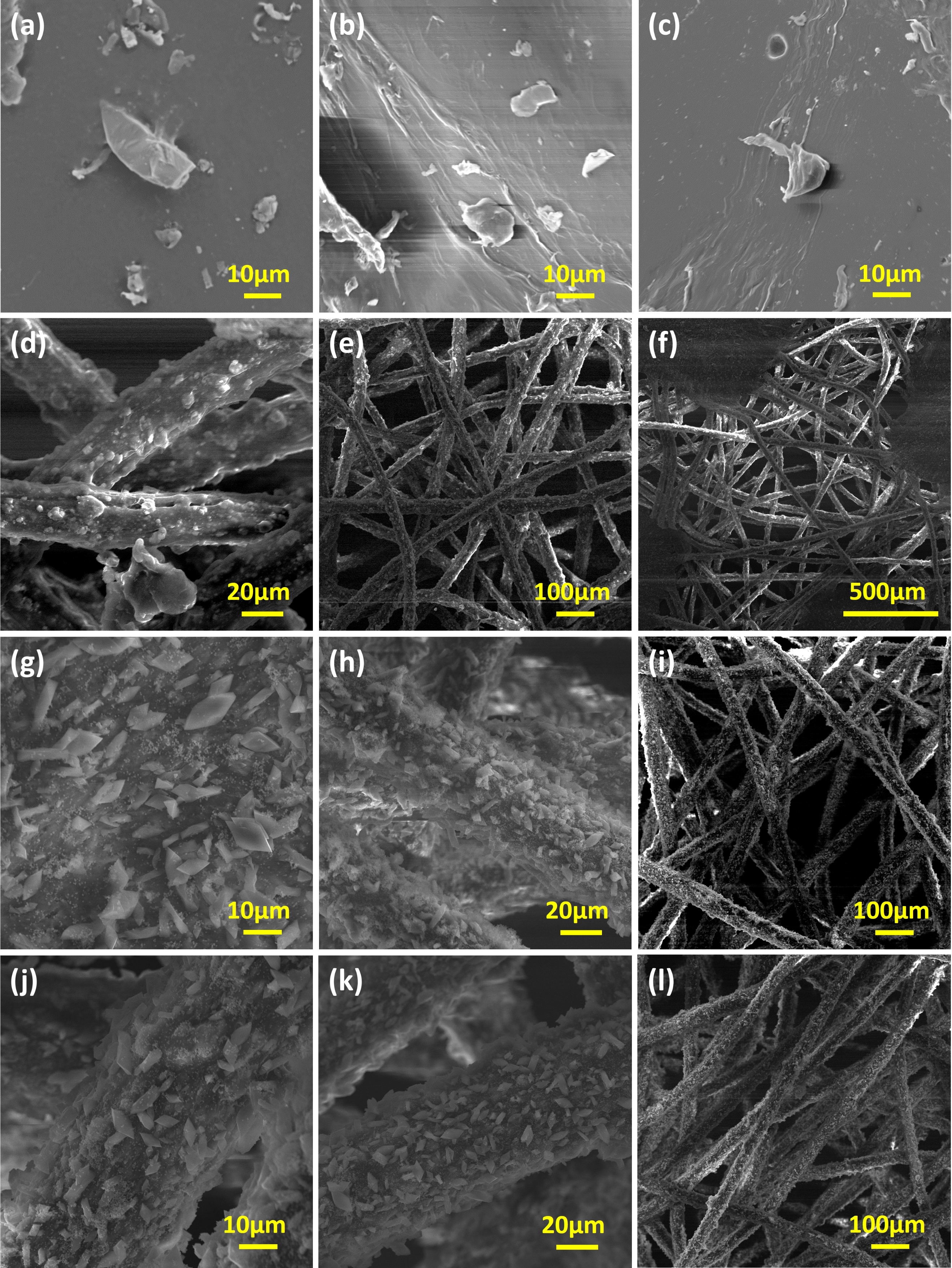


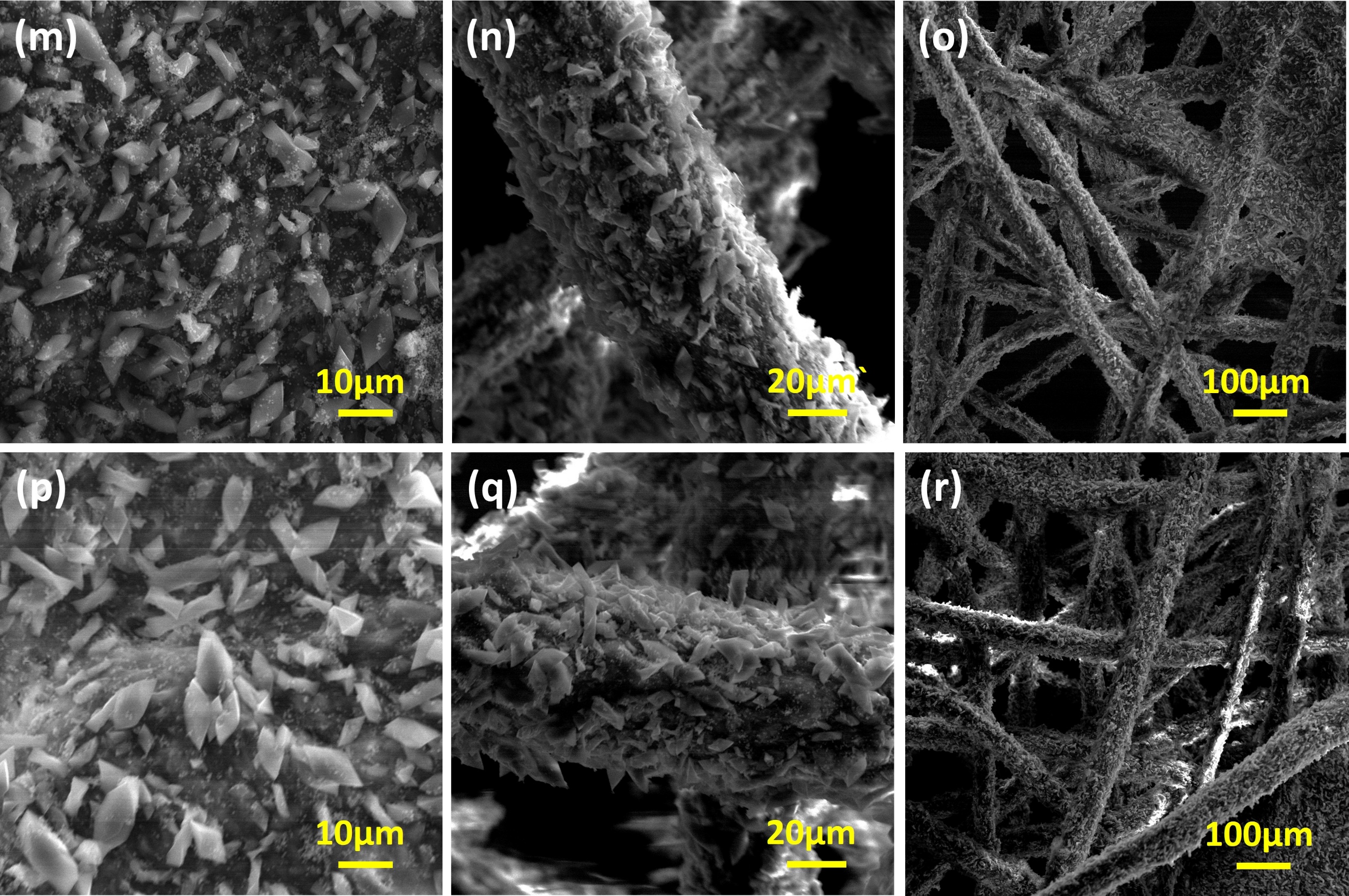


**Figure S2. Morphological characterization by SEM.** The SEM images showcase the structural morphologies of **(a-c)** PET-MPs, **(d-f)** bare PVDF-based UF membrane, and the synthesized Fe-MOF@UF membranes: **(g-i)** MU_1_ membrane prepared with the molar proportion of 1:1:1:500, **(j-l)** MU_2_ membrane fabricated with the molar ratio of 2:2:1:500, **(m-o)** MU_3_ membrane prepared with the molar ratio of 3:3:1:500, and **(p-r)** MU_4_ membrane synthesized with the molar ratio of 4:4:1:500.

**
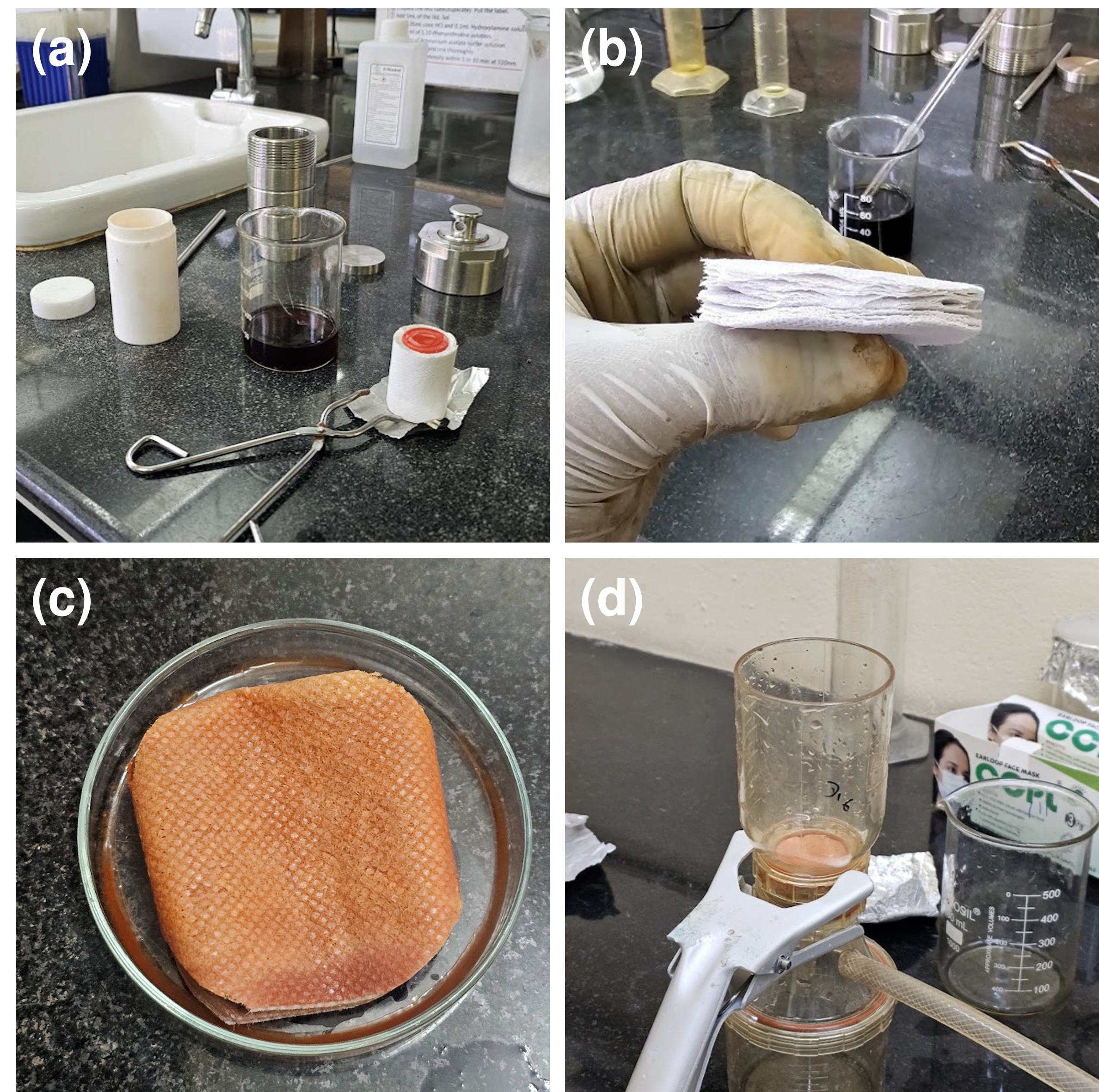
**

**Figure S3. Laboratory experimental images of MOF-incorporation onto UF membrane.** The captured images depict: (a) precursor solution containing FeCl_3_.6H_2_O, NH_2_-BDC, DI water, and DMF in desired proportions, along with commercially available cylindrical core UF membrane (white colored) beside; (b) vertically stacked membrane units prepared by cutting the cylindrical UF membrane filter to ensure fit within the laboratory vacuum filtration setup; (c) reddish-brown NH_2_-MIL-101(Fe) MOF deposited over membrane post-solvothermal synthesis; (d) vacuum filtration setup, with the MOF-integrated membrane sandwiched between filtering funnel and flask for further PET-MP removal analysis.

**
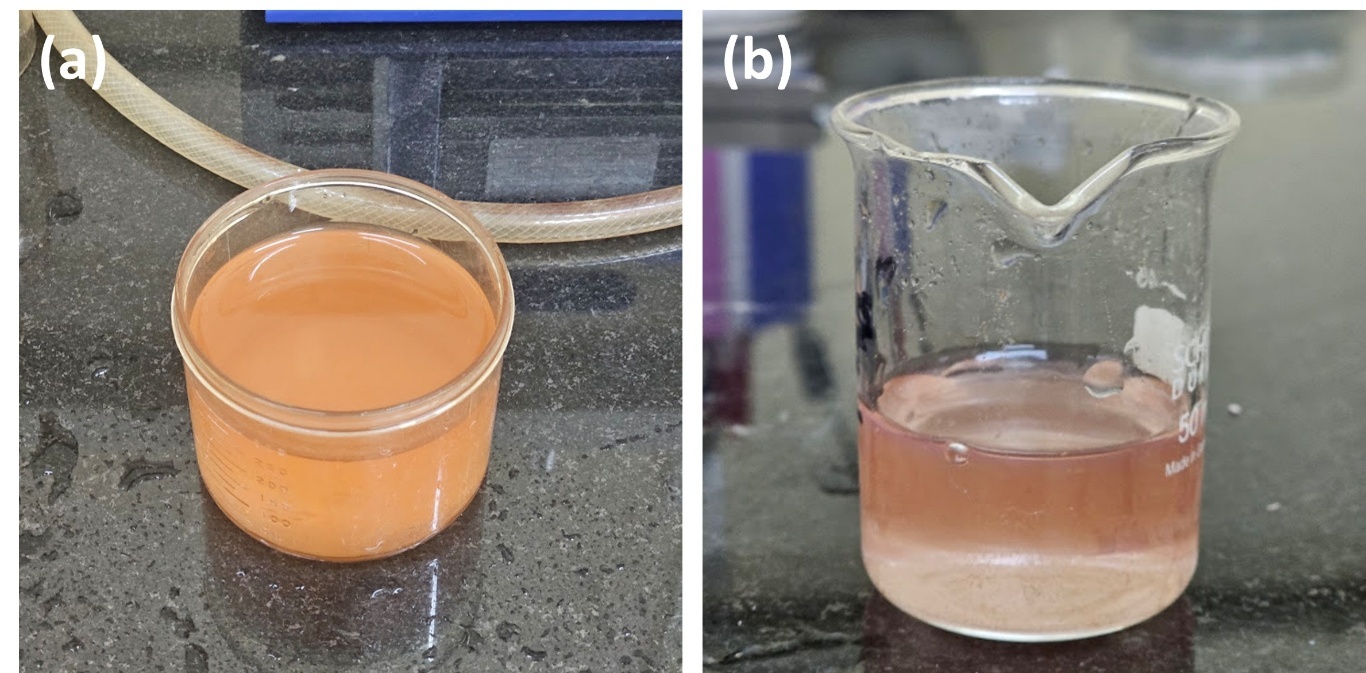
**

**Figure S4.** **Visual evidence of reddish coloration in the filtrate**. Photographs indicate the leaching of Fe-MOF particles from **(a)** MU_4_ membrane into the filtrate after the filtration, and **(b)** MU_3_ membrane synthesized at 140 ºC. Both conditions led to the rejection of these synthesis conditions, adopted for the fabrication of the respective membranes, for any further application.


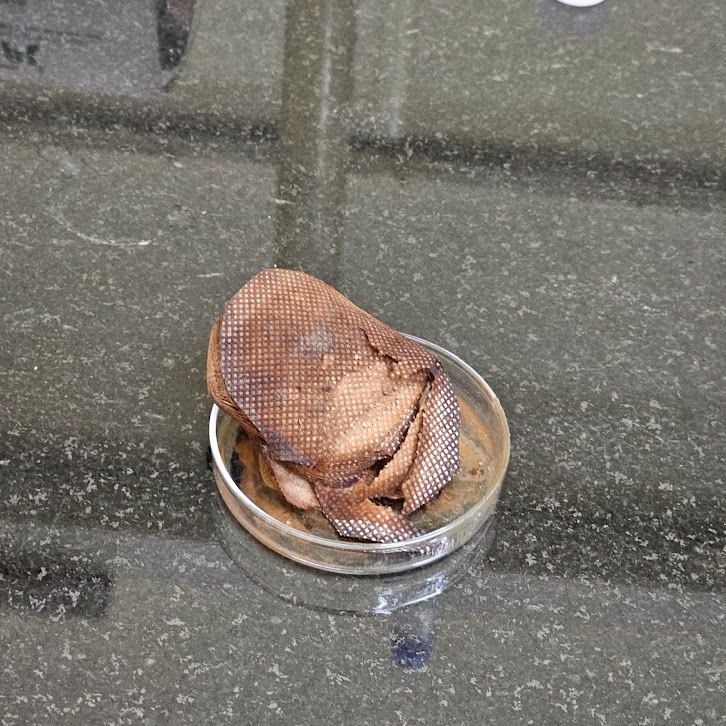


**Figure S5. Overburnt MU_3_ membrane.** Picture depicts the damages on the surface of the MU_3_ membrane surface caused by over-burning during the synthesis of the optimally loaded Fe-MOF@UF membrane at 160 ºC.

*
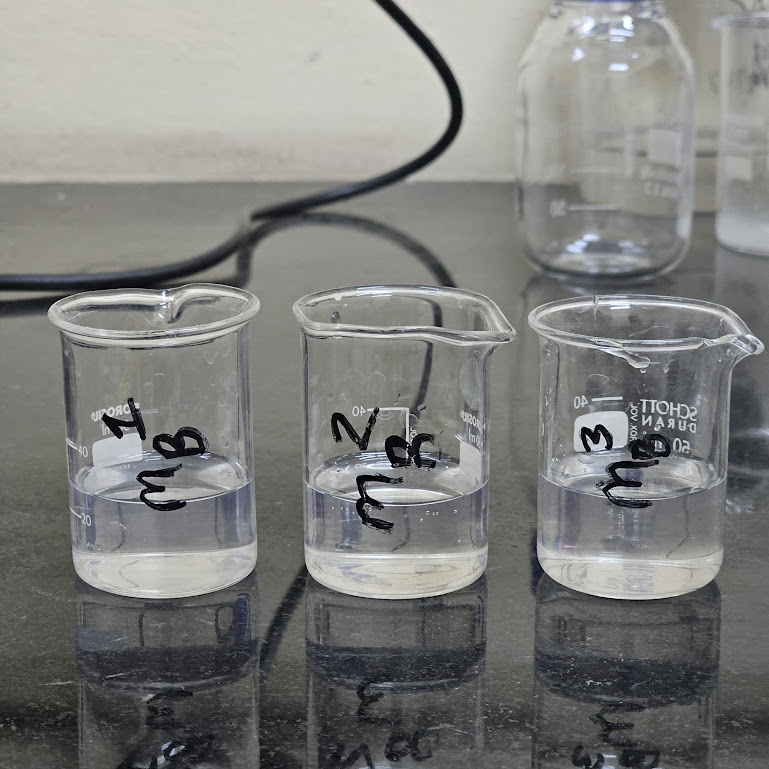
*

**Figure S6.** **Filtrates collected from the 4^th^ filtration cycle.** Photographs illustrate triplicate samples of filtrate obtained during the 4^th^ filtration cycle using the optimized Fe-MOF@UF membrane system. The triplicates were utilized to determine the removal efficiency of the membrane on 4^th^ consecutive cycle. Likewise, filtrates exhibit no visible discoloration or signs of MOF-leaching and were further analyzed for water quality to assess the presence of any chemical constituents indicative of MOF release.

**Table S1.** Data comprising of primary adsorption peaks obtained in FTIR spectral analysis and their respective nature of vibrations, necessary to identify the type of polymeric structure.

| Material type | Adsorption peaks (cm^-1^) | Nature of vibration | References |
| --- | --- | --- | --- |
| PET-MPs | 2970 - 2929 | C-H stretching | [10, 11] |
|  | 1714 | C=O stretching |  |
|  | 1240-1096 | C=O bond elongation and vibration |  |
|  | 872-723 | Swaying of glycol and out-plane benzene group |  |
| Bare PVDF UF membrane | 1427-1375 | In-plane -CH_2_ bending | [12, 13] |
|  | 1170 | Symmetrical -CF_2_ stretching |  |
|  | 972 | α-crystalline phase of PVDF |  |
|  | 844 | Common β- or γ-crystalline phase of PVDF |  |
| Fe-MOF@UF membrane | 3345 | Primary amine -NH_2_ bond vibration | [3, 14] |
|  | 1658 | C=N stretching vibration |  |
|  | 1578-1377 | Asymmetric and symmetric C=O vibrations |  |
|  | 1255 | Aromatic C-N stretching |  |
|  | 767-559 | O-Fe-O and Fe-O vibration modes |  |

**Table S2.** Specifications, composition details, and corresponding PET-MP rejection efficiencies of the Fe-MOF@UF membranes integrated with different MOF loadings.

| Membrane name | Dose-specific abbreviated names | Molar composition | MOF loading  (% by weight) | % PET-MP rejection efficiency |
| --- | --- | --- | --- | --- |
| Fe-MOF@UF | MU_0_ (Control) | – | 0 | 82.9 ± 2.4 |
|  | MU_1_ | 1:1:1:500 | 4 ± 0.1 | 85.6 ± 3.1 |
|  | MU_2_ | 2:2:1:500 | 8.5 ± 0.5 | 90.3 ± 0.8 |
|  | MU_3_ | 3:3:1:500 | 17.9 ± 1.4 | 94.2 ± 1.2 |
|  | MU_4_ | 4:4:1:500 | 21.7 ± 1.8 | 72.5 ± 9.3 |

**Table S3.** MOF loadings and corresponding PET-MP removal efficiencies for optimally loaded Fe-MOF@UF membranes, synthesized at different temperatures.

| Membrane | Synthesis Temperature (ºC) | MOF loading  (% by weight) | % PET-MP rejection efficiency |
| --- | --- | --- | --- |
| MU_3_ | 100 | 11.9 ± 1.2 | 91.4 ± 1.3 |
|  | 120 | 17.9 ± 1.4 | 94.2 ± 1.2 |
|  | 140 | 22.7 ± 0.6 | 86.1 ± 2.1 |
|  | 160 | (Overburnt) | |

**Table S4.** MOF loadings and corresponding PET-MP removal efficiencies for optimally loaded Fe-MOF@UF membranes, fabricated with different number of layers.

| Membrane | Number of membrane layers | MOF loading  (% by weight) | % PET-MP rejection efficiency |
| --- | --- | --- | --- |
| MU_3_ | 10 | 11.8 ± 1 | 89.3 ± 2.3 |
|  | 20 | 14.5 ± 0.4 | 91.6 ± 0.8 |
|  | 30 | 17.9 ± 1.4 | 94.2 ± 1.2 |

**Table S5.** Variations in PET-MP removal efficiencies of the optimally synthesized Fe-MOF@UF membrane system (MU_3_ membrane) when filtering feed solutions at varying pH levels.

| Membrane | pH | MOF loading  (% by weight) | % PET-MP rejection efficiency |
| --- | --- | --- | --- |
| MU_3_ | 5 | 17.7 ± 0.7 | 93.3 ± 1.5 |
|  | 7 | 17.9 ± 1.4 | 94.2 ± 1.2 |
|  | 9 | 17.7 ± 0.9 | 96.6 ± 1 |

**Table S6.** Variation in PET-MP removal efficiencies of the optimally synthesized Fe-MOF@UF membrane system (MU_3_ membrane) in the presence of ions in the feed solutions.

| Membrane | Ions | % PET-MP rejection efficiency |
| --- | --- | --- |
| MU_3_ | Calcium (Ca^2+^) | 95.9 ± 1 |
|  | Chloride (Cl^–^) | 89.2 ± 0.8 |
|  | Nitrate (NO_3_^–^) | 93.2 ± 1.4 |
|  | Sulphate (SO_4_^2–^) | 90.8 ± 1.6 |

**Table S7.** Differences in PET-MP removal efficiencies of the optimally synthesized Fe-MOF@UF membrane system (MU_3_ membrane) across two subsequent filtration cycles influenced by various desorbing agents.

| Membrane | Desorbing agent | Filtration cycle | % PET-MP rejection efficiency |
| --- | --- | --- | --- |
| MU_3_ | DI H_2_O (Control) | 1st | 94.1 ± 0.6 |
|  |  | 2nd | 90.5 ± 0.4 |
|  | Methanol | 1st | 93.9 ± 0.6 |
|  |  | 2nd | 84.3 ± 1 |
|  | Ethanol | 1st | 94.4 ± 1.5 |
|  |  | 2nd | 87.1 ± 1.4 |
|  | 0.1M NaOH | 1st | 94 ± 0.9 |
|  |  | 2nd | 91.4 ± 1.1 |

**Table S8.** Declination in PET-MP rejection efficiencies of the optimally synthesized Fe-MOF@UF membrane system (MU_3_ membrane) across multiple filtration cycles while using 0.1M NaOH as the optimal desorbing agent.

| Membrane | Desorbing agent | Filtration cycle | % PET-MP rejection efficiency |
| --- | --- | --- | --- |
| MU_3_ | 0.1M NaOH | 1st | 94 ± 0.9 |
|  |  | 2nd | 91.4 ± 1.1 |
|  |  | 3rd | 89.8 ± 0.4 |
|  |  | 4th | 88.4 ± 0.6 |
|  |  | 5th | 86.2 ± 1.4 |
|  |  | 6th | 85.2 ± 0.2 |
|  |  | 7th | 84.1 ± 0.3 |
|  |  | 8th | 82.5 ± 0.9 |
| MU_0_ (Control) | – | 1st | 82.9 ± 2.4 |

**Table S9.** Analysis of the water quality parameters of the 4^th^-cycle filtrate (selected as a representative mid-cycle condition) obtained through the optimized Fe-MOF@UF membrane.

| Water quality parameters | Test values |
| --- | --- |
|  |  |
| pH | 7.37 ± 0.09 |
| Conductivity (µS/cm) | 411 ± 14.34 |
| TDS (mg/L) | 203 ± 11.10 |
| Turbidity (NTU) | 2.03 ± 0.3 |
| Color (TCU) | BDL ^a)^ |
| DO (mg/L) | 5.18 ± 0.21 |
| Iron (mg/L) | ND ^b)^ |
| Ammonia (mg/L) | ND ^b)^ |

^a)^ Below Detection Limit (BDL); ^b)^ Not Detected (ND).

**Table S10.** Validating the MP removal efficiency of the optimized Fe-MOF@UF membrane system in removing MPs from real, commercial, PET-bottled drinking water samples.

| Membrane | Initial MP concentration  (particles/L) | Final MP concentration  (particles/L) | MP rejection efficiency  (%) |
| --- | --- | --- | --- |
| Fe-MOF@UF (optimized)  [Molar proportion = 3:3:1:500;  Synthesis temperature = 120 ºC;  Number of membrane layers = 30] | 70 ± 12.2 | 8.3 ± 8.5 | 89.1 ± 9.7 |

**Table S11.** Analysis of the key water quality parameters of the filtrate obtained from the filtration of sampled drinking water through the optimized Fe-MOF@UF membrane.

| Water quality parameters | Test values | Maximum Concentration Limits | | Ref. |
| --- | --- | --- | --- | --- |
|  |  | WHO ^a)^ | EPA ^b)^ |  |
| pH | 7.26 ± 0.12 | NA ^d)^ | 6.5 – 8.5 ^c)^ | [6, 7] |
| Conductivity (µS/cm) | 465 ± 32.57 | NA ^d)^ | NA ^d)^ |  |
| TDS (mg/L) | 301 ± 18.67 | 1000 | 500 |  |
| Turbidity (NTU) | 3.17 ± 0.8 | 5 | 5 |  |
| Color (TCU) | BDL ^e)^ | 15 | 15 |  |
| DO (mg/L) | 6.34 ± 0.32 |  |  |  |
| Total Hardness as CaCO_3_ (mg/L) | 306.67 ± 33.99 | 500 | NA ^d)^ |  |
| Chloride (mg/L) | 108.87 ± 17.71 | 250 | 250 |  |
| Iron (mg/L) | ND ^f)^ | 0.3 | 0.3 |  |
| Arsenic (mg/L) | ND ^f)^ | 0.01 | 0.01 |  |
| Ammonia (mg/L) | 0.2 ± 0.08 | NA ^d)^ | NA ^d)^ |  |
| Nitrate (mg/L) | 1 ± 0.47 | 50 | 10 |  |
| Manganese (mg/L) | 0.03 ± 0.01 | 0.08 | 0.05 |  |
| Copper (mg/L) | BDL ^e)^ | 2 | 1 |  |
| Zinc (mg/L) | 0.11 ± 0.02 | 3 | 5 |  |

^a)^ Guidelines for drinking-water quality, 2022, World Health Organization (WHO);
^b)^ Drinking Water Standards and Health Advisories Tables, 2018, United States Environmental Protection Agency (USEPA); ^c)^ These values show lower and upper limit.;
^d)^ Not Available (NA); ^e)^ Below Detection Limit (BDL); ^f)^ Not Detected (ND).

**References**

1. Y. J. Chen, Y. Chen, C. Miao, et al., “Metal-organic framework-based foams for efficient microplastics removal,” *Journal of Material Chemistry A* 8, (2020): 14644–14652. <https://doi.org/10.1039/d0ta04891g>

2. X. Liu, N. K. Demir, Z. Wu, K. Li, “Highly Water-Stable Zirconium Metal-Organic Framework UiO-66 Membranes Supported on Alumina Hollow Fibers for Desalination,” *Journal of the American Chemical Society* 137, (2015): 6999–7002. <https://doi.org/10.1021/jacs.5b02276>

3. L. K. Njaramba, Y. Yoon, C. M. Park, “Fabrication of porous beta-cyclodextrin functionalized PVDF/Fe–MOF mixed matrix membrane for enhanced ciprofloxacin removal,” *Npj Clean Water* 7, (2024). <https://doi.org/10.1038/s41545-024-00312-x>

4. J. Zhang, M. Peng, E. Lian, et al., “Identification of Poly(ethylene terephthalate) Nanoplastics in Commercially Bottled Drinking Water Using Surface-Enhanced Raman Spectroscopy,” *Environmental Science & Technology* 57, (2023): 8365–8372. <https://doi.org/10.1021/acs.est.3c00842>

5. B. E. Oßmann, G. Sarau, H. Holtmannspötter, “ Small-sized microplastics and pigmented particles in bottled mineral water,” *Water Research* 141, (2018): 307–316. <https://doi.org/10.1016/j.watres.2018.05.027>

6. U.S. Environmental Protection Agency, “2018 Edition of the Drinking Water Standards and Health Advisories Tables,” Office of Water, U.S. Environmental Protection Agency, Washington, DC (2018). <https://www.epa.gov/system/files/documents/2022-01/dwtable2018.pdf/>

7. World Health Organization, “Guidelines for drinking-water quality, 4th edition, incorporating the 1st addendum,” World Health Organization (2022). <https://iris.who.int/bitstream/handle/10665/254637/9789241549950-eng.pdf?sequence=1>

8. S. Modak, M. Kasula, M. R. Esfahani, “Nanoplastics Removal from Water using Metal–Organic Framework: Investigation of Adsorption Mechanisms, Kinetics, and Effective Environmental Parameters,” *ACS Applied Engineering Materials* 1, (2023): 744–755. <https://doi.org/10.1021/acsaenm.2c00174>

9. J. Masura, J. Baker, G. Foster, C. Arthur, “Laboratory Methods for the Analysis of Microplastics in the Marine Environment: Recommendations for quantifying synthetic particles in waters and sediments,” U.S. Department of Commerce, National Oceanic and Atmospheric Administration (NOAA) (2015). <https://repository.library.noaa.gov/view/noaa/10296>

10. C. Ioakeimidis, K. N. Fotopoulou, H. K. Karapanagioti, et al., “The degradation potential of PET bottles in the marine environment: An ATR-FTIR based approach,” *Scientific Rep*orts 6, (2016): 1–8. <https://doi.org/10.1038/srep23501>

11. S. Kauts, Y. Mishra, S. Yousuf, et al., “Toxicological Profile of Polyethylene Terephthalate (PET) Microplastic in Ingested Drosophila melanogaster (Oregon R+) and Its Adverse Effect on Behavior and Development,” *Toxics* 11, (2023). <https://doi.org/10.3390/toxics11090782>

12. S. Mohamadi, “Preparation and Characterization of PVDF/PMMA/Graphene Polymer Blend Nanocomposites by Using ATR-FTIR Technique,” *Infrared Spectroscocpy - Material Science, Engineering and Technology* (2012). <https://doi.org/10.5772/36497>

13. Q. Ye, J. Xu, Y. Zhang, et al., “Metal-organic framework modified hydrophilic polyvinylidene fluoride porous membrane for efficient degerming selective oil/water emulsion separation,” *Npj Clean Water* 5, (2022): 1–9. <https://doi.org/10.1038/s41545-022-00168-z>

14. Q. Xie, Y. Li, Z. Lv, et al., “Effective Adsorption and Removal of Phosphate from Aqueous Solutions and Eutrophic Water by Fe-based MOFs of MIL-101,” *Scientific Reports* 7, (2017): 1–15. <https://doi.org/10.1038/s41598-017-03526-x>
